# H3K27 acetylation activated-COL6A1 promotes osteosarcoma lung metastasis by repressing STAT1 and activating pulmonary cancer-associated fibroblasts

**DOI:** 10.7150/thno.51245

**Published:** 2021-01-01

**Authors:** Ying Zhang, Zhaoyong Liu, Xia Yang, Weiqing Lu, Yelong Chen, Youbin Lin, Jin Wang, Suxia Lin, Jing-Ping Yun

**Affiliations:** 1 Sun Yat-sen University Cancer Center; State Key Laboratory of Oncology in South China; Collaborative Innovation Center for Cancer Medicine, Guangzhou 510060, China.; 2 Department of Pathology, Sun Yat-sen University Cancer Center, Guangzhou 510060, China.; 3 Department of Orthopedics, First Affiliated Hospital of Shantou University Medical College, No.57 Changping Road, Shantou, Guangdong 515041, China.; 4 Department of Orthopedics, Sun Yat-sen University Cancer Center, Guangzhou 510060, China.

**Keywords:** COL6A1, osteosarcoma, STAT1, metastasis, cancer-associated fibroblasts

## Abstract

**Background:** Collagen type VI alpha 1 (COL6A1) has been found to be dysregulated in several human malignancies. However, the role of COL6A1 in osteosarcoma (OS) progression remains largely unclear. Here, we aimed to explore the clinical significance and biological involvement of COL6A1 in the OS cell migration and invasion.

**Material and Methods:** We used immunohistochemistry, qRT-PCR and western blot to detect the expression of COL6A1 in 181 OS patient samples. Chromatin immunoprecipitation (ChIP) and PCR were carried out to verify the regulatory interaction of p300, c-Jun and COL6A1 promoter. The invasion and migration function of COL6A1 in OS was detected *in vitro* and *in vivo*. RNA sequence was performed to detect the downstream pathway of COL6A1, and then co-immunoprecipitation (co-IP), ubiquitination assays and rescue experiments were performed to determine the regulatory effect of COL6A1 and signal transducers and activators of transcription (STAT1). Exosomes derived from OS cell lines were assessed for the ability to promote cancer progression by co-cultured assay and exosomes tracing.

**Results:** COL6A1 was commonly upregulated in OS tissues, especially in lung metastasis tissues, which was associated with a poor prognosis. c-Jun bound p300 increased the enrichment of H3K27ac at the promoter region of the COL6A1 gene, which resulted in the upregulation of COL6A1 in OS. Overexpression of COL6A1 promoted OS cell migration and invasion via interacting with SOCS5 to suppress STAT1 expression and activation in an ubiquitination and proteasomal degradation manner. Most interestingly, we found that exosomal COL6A1 derived from OS cells convert normal fibroblasts to cancer-associated fibroblasts (CAFs) by secreting pro-inflammatory cytokines, including IL-6 and IL-8. The activated CAFs could promote OS cell invasion and migration by mediating TGF-β/COL6A1 signaling pathway.

**Conclusion:** Our data demonstrated that upregulation of COL6A1 activated by H3K27 acetylation promoted the cell migration and invasion by suppressing STAT1 pathway in OS cells. Moreover, COL6A1 can be packaged into OS cell-derived exosomes and activate CAFs to promote OS metastasis.

## Introduction

Osteosarcoma is the most common primary malignant bone tumor that frequently occurs in children and adolescents, has a strong tendency to metastasize 1. Pulmonary metastasis accounts for 90% of the distant metastases and is the main cause of treatment failure and death in patients with OS 2. The 5-year survival rate has remained at 60-70% among patients with localized tumors, but this is only 20% in patients who had lung metastasis 3. Thus, there is an urgent need to investigate the molecular mechanisms underlying OS metastasis and develop effective approaches to suppress lung metastasis.

Collagen type VI alpha 1 (COL6A1) belongs to a family of collagens that are widely distributed in several tissues, including the skeletal muscle, skin, lungs, and blood vessels 4. The *COL6A1* gene is located at chromosome 21q22.3 and encodes the alpha 1 chain of type VI collagen, which is a component of microfibrillar structures 5. The COL6A1 has one N-terminal and two C-terminal von Willebrand factor type A (vWFA) modules 4. The COL6A1 protein is known to be localized in the extracellular matrix and is involved in cell adhesion and collagen remodeling 6. Emerging evidence has demonstrated that COL6A1 has diverse biological functions, including cell migration, differentiation, embryonic development, and maintenance of cell stemness 4. There are a few studies regarding the expression and role of COL6A1 in human malignancies, although high expression of COL6A1 observed in prostate cancer, renal cell carcinoma, and cervical cancer was associated with poor survival outcome 7-9. Furthermore, overexpression of COL6A1 enhances pancreatic cancer cell motility and metastasis, while COL6A1 knockdown leads to suppression of this metastatic ability 8. However, there are no studies regarding the potential role of COL6A1 in the biological processes leading to OS development and progression.

Cancer-associated fibroblasts (CAFs) are activated fibroblasts that reside within the tumor microenvironment (TME) and are one of the most prominent cell types in the stroma, where they produce large amounts of extracellular matrix molecules, chemokines, cytokines, and growth factors 10. However, CAFs are very heterogeneous and express different specific markers that can be used for identification. The most used markers for CAFs are α-smooth muscle actin, fibroblast activation protein, and vimentin 11. Moreover, CAFs are thought to promote tumor cell growth, invasion, metastasis, drug resistance, and stemness 12. In the TME, CAFs build up and remodel the extracellular matrix, which enables the tumor cells to invade through the TME and interact with other cancer cells or stromal cells 10. This interaction involves the secretion of growth factors, cytokines, and chemokines, such as interleukin (IL)-1β, IL-6, IL-8, transforming growth factor-β (TGF-β), and collagen 13, although the interaction of OS cells with CAFs remains to be studied in details.

Exosomes are important mediators of intercellular communication, which contain microRNAs (miRNAs), mRNAs, DNA fragments, and proteins in small vesicular bodies (diameter: 30-100 nm) 14. Exosomes mediate cell-to-cell communication and activate signaling pathways by delivering their contents from donor cells to target cells. Accumulating evidence also suggests that exosomes are involved in the regulation of cell proliferation, differentiation, metabolism, and apoptosis 15-17. Specific exosomes released from CAFs can be internalized by cancer cells and contribute to progression and metastasis by transferring various substances 18. Thus, exosomes secreted from tumor cells may facilitate tumor invasion and metastasis by converting fibroblasts into CAFs and promoting the development of a TME that supports tumor cell proliferation, angiogenesis, invasion, and pre-metastatic niche preparation 19.

The present study revealed that COL6A1 was significantly upregulated in OS tissues and was related to lung metastasis and poor survival among patients with OS. The c-Jun transcription factor bound p300 and increased the enrichment of H3K27ac at the promoter region of the COL6A1 gene, which resulted in the upregulation of COL6A1 in OS tissues. Furthermore, COL6A1 downregulated STAT1 by promoting its ubiquitination and proteasomal degradation, which promoted OS metastasis. Moreover, COL6A1 could be packaged into exosomes and transferred from OS cells to fibroblasts, which drive conversion into activated CAFs. The activated CAFs could promote OS cell invasion and migration by secreting TGF-β. Therefore, we found that COL6A1 promoted OS metastasis by suppressing STAT1 expression and activating CAFs.

## Materials and Methods

### Patient samples and cell lines

This study included 181 randomly selected patients with primary OS who underwent radical resection at the First Affiliated Hospital of Shantou University Medical College, First Affiliated Hospital of Zhejiang University Medical College, First Affiliated Hospital of Sun Yat-sen University Medical College and Sun Yat-sen University Cancer Center from 2010 to 2019. None of the patients received preoperative radiotherapy or chemotherapy (**Table S1**). OS samples (n = 181), non-tumor tissues (n = 44) and lung metastasis tissues (n = 9) were used to analyze COL6A1 protein expression by immunohistochemistry. 181 OS cases included tissues with metastatic (n = 29) and without metastatic (n = 152). 98/181 (54%) were male and 83/181 (46%) were female. The median age was 24.5 years (range 8-79 years). 41 pair of case-matched OS and non-tumor tissues were obtained to evaluate COL6A1 expression by western blot and 22 fresh OS tissues and 7 non-tumor tissues were used to analyze COL6A1 expression at mRNA level. Human serum specimens were collected from healthy donors and OS patients before resection in the Sun Yat-sen University Cancer Center. The serum samples included 10 healthy controls, 25 OS patients without lung metastasis, and 5 OS patients suffering lung metastasis. This study was approved by the ethical review committees of the Sun Yat-sen University Cancer Center. All participants involved in our study provided written informed consents.

Three human OS cell lines MG63, U2OS and Saos-2 were maintained in Dulbecco's modified Eagle's medium (DMEM) (Gibco, Waltham, MA, USA) supplemented with 10% fetal bovine serum (Gibco) and 1× antibiotic mixture (Invitrogen, Carlsbad, CA, USA). Osteoblast cell line hFOB1.19 was purchased from American Type Culture Collection (Manassas, VA, USA). MRC5 was purchased from Sciencell Company (USA) and cultured in Eagle's minimum essential medium (Gibco) supplemented with 10% FBS (Gibco). Cell lines were authenticated by short tandem repeats (STR) profiling and confirmed to be mycoplasma negative.

### Drugs, siRNA and Plasmid

Proteasome inhibitor MG132, histone deacetylase (HDAC) inhibitor NaBu, p300 inhibitor C646 and A485, Src inhibitor were purchased from MCE company (NJ, USA). Cycloheximide (CHX) was purchased from Sigma-Aldrich (St. Louis, MO, USA). The siRNAs targeting indicated genes were conducted by Gemepharma Company (Shanghai, China).

The expression vectors containing the p300, c-Jun, COL6A1, SOCS5, mutant SOCS5, HA-tagged Wild type (wt) Ubiqution (Ub), HA-tagged K11R Ub, HA-tagged K48R Ub and HA-tagged K63R Ub plasmids were purchased from Vigene (Shanghai, China). GFP-tagged wild type STAT1 (wt), GFP-tagged STAT1 (Y701A), and GFP-tagged STAT1 (S727A) were purchased from Addgene (Watertown, MA, USA).

### Establishment of stable overexpression or knock-down cell lines

For overexpression of COL6A1 or STAT1 in OS cell lines, the full-length cDNA encoding COL6A1 was subcloned into the pENTER vector (Invitrogen). After virus packaged in HEK293T cells using Lipofectamine 3000, COL6A1, STAT1 or mock vectors were transfected into target cell lines. For knockdown of COL6A1 or STAT1, shRNA sequences targeting was cloned into a pLKO.1-TRC cloning vector. ShRNA-containing plasmids were packaged and transfected into target cell lines. Transfected cells used for overexpression or knockdown were selected with puromycin (5 µg/mL) for 2 weeks.

### Western blot analysis

Western blot assay was performed as described previously 20. Briefly, total protein from cells was extracted using RIPA buffer (Beyotime, Shanghai, China) supplemented with complete EDTA-free Protease Inhibitor Cocktail (Roche Diagnostics, Basel, Switzerland). The extracted protein was separated on SDS polyacrylamide gels and transferred to the PVDF membranes. Western blotting was performed using antibodies against human COL6A1 (Abcam, Milton, Cambridge, UK), N-cadherin, E-cadherin, Vimentin, FAK, Src, p-FAK, p-Src, p-STAT1 (Tyr701), p-STAT1 (Ser727), p-STAT3 (Tyr705), STAT1, STAT3, CD133, ABC2G, SOX2, Nanog, CD63, CD9, TSG101, SOCS5, Flag, GFP, HA, Ubiqution (Ub), GAPDH, p300, c-Jun were purchased from Cell Signaling Technology (CST, USA).

### Immunohistochemistry (IHC)

IHC staining was performed using the Envision Labeled Peroxidase System (Dako, Carpinteria, CA) as described previously 20. IHC staining of COL6A1 and STAT1 was examined by two pathologists. Expression of COL6A1 and STAT1 were analyzed by an individual labeling score considering the proportion of positively stained tumor cells and the intensity of staining. Intensity of stained cells was graded into four levels: 0: negative staining; 1: weak staining; 2: mild staining; and 3: strong staining. The area of staining was evaluated and recorded as a percentage: 0: no staining; 1: positive staining in 1 to 25% of tumor cells; 2: 26% to 50%; 3: 51% to 75%; 4: > 76% of tumor cells. Intensity and fraction of positive cell scores were multiplied and thus the scoring system was defined as low expression for scores of 0-6, and high expression was defined when the score is 8-12.

### RNA extraction and Quantitative RT-PCR

RNA was extracted from the OS cells by RNAeasy^TM^ Plus Animal RNA Isolation Kit with Spin Column (Beyotime, Jiangsu, China). cDNA was synthesized with SuperScript III (Takara, Dalian, China) according to the manufacturer's protocol. Real-time PCR analysis was performed using the Applied Biosystems 7500 Real-Time PCR System, according to the manufacturer's instructions. The reactions were performed for three independent experiments and results were normalized to β-actin. The primer sequences used can be found in the **Table S2**. The mean±SD of three independent experiments is shown.

### RNA sequence

Total RNA was extracted using Trizol reagent kit (Invitrogen, Carlsbad, CA, USA) according to the manufacturer's protocol. RNA quality was assessed on an Agilent 2100 Bioanalyzer (Agilent Technologies, Palo Alto, CA, USA) and checked using RNase free agarose gel electrophoresis. After total RNA was extracted, eukaryotic mRNA was enriched by Oligo(dT) beads, while prokaryotic mRNA was enriched by removing rRNA by Ribo-ZeroTM Magnetic Kit (Epicentre, Madison, WI, USA). Then the enriched mRNA was fragmented into short fragments using fragmentation buffer and reverse transcripted into cDNA with random primers. Second-strand cDNA were synthesized by DNA polymerase I, RNase H, dNTP and buffer. Then the cDNA fragments were purified with QiaQuick PCR extraction kit (Qiagen, Venlo, The Netherlands), end repaired, poly(A) added, and ligated to Illumina sequencing adapters. The ligation products were size selected by agarose gel electrophoresis; PCR amplified, and sequenced using Illumina Novaseq 6000 by Gene Denovo Biotechnology Co. (Guangzhou, China).

### Chromatin immunoprecipitation (ChIP) assays

ChIP assays were performed using an EZ-Magna ChIP kit (Millipore,Billerica, MA, USA). ChIP assays were essentially performed as protocol described with slight modifications: For OS tissues and case matched non-tumor (bone) tissues, weigh fresh frozen tissue and cut it into 1-3 mm^3^ pieces. Then grind in liquid nitrogen and add 10 mL of 1X PBS. Add formaldehyde until the final concentration is 1%. Rotate for 15-20 min at room temperature. Add 2.5 m Glycine to the final concentration of 0.125 m then rotate 10 mins and centrifuge. Wash with 1X PBS and remove the supernatant. Add 2 mL of frozen 1X PBS. The homogenizer cracked the tissue with the ChIP lysis buffer with protease inhibitor and incubated for 10-15 mins on ice, then centrifuged and flowed, following the manufacturer's protocol. For OS cells MG63 and osteoblast cell line hFOB1.19, 5×10^6^ cells were fixed with formaldehyde (1% final volume concentration, Sigma) for 10 min at room temperature. Fixation was stopped with the addition of 1/10 volume 1.25 M glycine and the samples were incubated for 5 min at room temperature. The sonication step was performed in a Covaris sonicator (5 min, 20 s on, 20 s off), and 200 µg of the protein-chromatin complex was used in each immunoprecipitation. Antibody-protein complexes were captured with preblocked dynabeads protein G (Invitrogen). ChIP DNA was analyzed by qPCR with SYBR Green (Takara) on an ABI-7500 (Applied Biosystems) using the primers specified in **Table S1**. The antibodies used are as followed: H3K27ac (CST), p300 (Santa Cruz Biotechnology, Santa Cruz, CA), and normal mouse IgG (Millipore).

### Co-immunoprecipitation (co-IP)

Co-IP was performed using indicated antibodies and IgG (Invitrogen) according to manufacturer's instruction. In brief, cell lysates were incubated with antibody-conjugated beads at 4 °C for 2 h. Then, the beads were washed extensively and boiled in SDS loading buffer. Western blot was used to study the immnuoprecipitated proteins.

### Cell proliferation assay

Cell viability was tested with Cell Counting Kit-8 (CCK8) kit (Sigma) according to the manufacturer's instruction. OS cells were trypsinized, counted and then plated at a density of 2×10^3^ cells per well in 96-well plates. The cells were incubated at 37 °C. The attached cells were added with CCK8 dilution for 1 h, and then the absorbance of each well of the plates was measured at 450 nm.

### Flow cytometry

The apoptosis assay was performed after transfection using the Annexin V-fluorescein isothiocyanate Apoptosis Detection Kit I (BD Biosciences, San Jose, CA, USA) and assessed by flow cytometry. CD133 antibodies were obtained from eBioscience (CA, USA). OS cells were incubated with CD133 antibodies (1:1000) in PBS containing 1% bovine for 30 min at 4 °C. After washing with PBS, the cells were analyzed by flow cytometry (Cyto FLEX, Beckman, Germany). The results were analyzed using Cyt Expert.

### Transwell assay

OS cells were seeded into transwell chambers (Millipore) in triplicate at a density of 5 × 10^4^ cells per well in 200 µL serum-free DMEM medium. Then 750 µL DMEM containing 10% FBS was added into the lower compartment of the transwell chamber. Cells could migrate for 24 h or invade through the Matrigel (BD Biosciences) for 48 h. Migrated cells were fixed with 4% paraformaldehyde and stained with 0.2% crystal violet, and then counted under a light microscope at a magnification of 200.

### Colony formation assay

After COL6A1 or empty vector transfection, cells were added after 500 cells/well were plated in six-well plates and incubated 10 days at 37 °C. The cells were fixed with 4% buffered formalin for 15 min and then stained with 1% crystal violet (Sigma Aldrich) for 30 min. The plates were gently washed with PBS and dried before microscopic evaluation. Cell clusters with >30 cells were considered as a colony.

### Cell adhesion assay

OS cells were trypsinized, counted and then plated at a density of 3 × 10^4^ cells per well in 96-well plates coated with collagen I, collagen IV, laminin or fibronectin (10 μg/mL). The cells were incubated at 37 °C for 1 h. The attached cells were fixed with 1% glutaraldehyde for 30 min and stained with 1% crystal violet in water for 30 min. After washing with distilled water, the stained cells were extracted with 0.1% Triton X-100. The absorbance of each well of the plates was measured at 570 nm.

### Anoikis Assay

To prevent cell adhesion, tissue culture six-well plates were coated with 200 μL (6 mg/mL in 95% ethanol) of 2-hydroxyethylmethacrylate (poly-HEMA) and left to temperature. 10^4^ Suspended cells were treated and incubated at 37 °C before harvested several days for cell viability test using MTT assay. Afterwards, 50 mL of 250 mg MTT was supplemented and incubated for 4 h at 37 °C. The precipitate was dissolved in cell lysis buffer (10% sodium dodecylsulfate, 0.01 mol/L HCl), and cell viability was determined by the absorbance value at 570 nm.

### Collagen contraction assays

A total of 2 × 10^5^ MRC5 were suspended in 100 μL DMEM. Then the cell suspension was mixed with 100 μL of collagen mix containing 68.75 µL DMEM, 0.72 µL 1 N NaOH, and 31.25 µL Type 1 Rat Tail Collagen (Corning, NY, USA), and added to 1 well of 24-well plates and allowed to solidify for 45 min at 37 °C. After incubation with media containing tumor-derived exosomes, the gels were photographed at different time points. Image J software was employed to measure gel area and evaluates contraction.

### Luciferase activity assay

Transcription activity was measured by a luciferase reporter gene assay using the Dual-Luciferase reporter assay system according to the manufacturer's instructions (Promega Corporation, Madison, WI, USA). Reporter gene expression was measured and quantified by using a dual Luciferase Reporter Assay System (Promega). The luciferase activity was normalized to the activity of the Renilla luciferase control. Extracts from each transfection were assayed in triplicate from at least three independent transfection experiments. The results were expressed as means ± SD.

### Subcellular fractionation and DNA binding assays

Subcellular fractionation and DNA binding assays were conducted. Nuclear and cytoplasmic protein of OS cells was extracted using an NE-PER protein extraction kit (Thermo Scientific, USA) according to the manufacturer's instructions. For western blot analysis, tubulin and histone deacetylase 1 (HDAC1) were used for the cytoplasmic and nuclear loading control, respectively.

Oligonucleotide pull-down assays were performed with an annealed nucleotide comprising the STAT1 consensus site (5′-CATGTTATGCATATTCCTGTAAGTG-3′) and STAT3 consensus site (5'-GATCCTTCTGGGAATTCCTAGATC-3') with a 5-biotin label. Nuclear extracts (50-100 μg) were incubated for 1 h at 4 °C with 1 μg oligonucleotide in binding buffer, and then Sepharose-streptavidin (50 μL; Sigma) was added for 2 h at 4 °C. After three washes in PBS, the complexes were suspended in SDS sample buffer and processed for western blotting, as described above, and probed with anti-STAT1, STAT3 antibodies (CST).

### ELISA

Conditioned media from cancer cells plated with same cell numbers were transfected with COL6A1 or control, harvested, and analyzed using the Human IL-6, IL-8 (Absion, China) and TGF-β quantikine ELISA kit (Novus, China), following the instructions provided by the manufacturer. Briefly, the 50 μL of sample and standard lysate were added to microplate wells respectively. Then 100 μL of detection reagent was added to each assay well. After incubation for 20 min, the absorbance at 450 nm was measured using an ELISA microplate reader.

### Culture medium protein extraction

The indicated OS cells at 80% confluence were incubated in serum-free DMEM for three days. The CM were collected, filtered, and then added 4 volumes of cold acetone. After mix and keep at -20 °C overnight. Then spin at maximum speed for 15 min at 4 °C and discharge supernatant and retain the pellet. Dry samples under vacuum and resuspend samples in a minimal volume of RIPA buffer. The expression of indicated protein was detected by western blot.

### Exosome isolation and identification

Exosomes were isolated from culture medium by using ExoQuick-TC (SBI, PA, USA). The purified exosomes were resuspended in PBS for functional assays or used for protein detection. The protein concentration of exosomes was determined using a BCA Protein Assay Kit (Beyotime). The size and quality of exosomes was determined using a LM10-HS NanoSlight instrument and nanoparticle tracking analysis (NTA) software and transmission electron microscope (TEM).

### Exosomes tracing

For exosome-tracing experiments, tumor cells were pre-treated by a PKH67 green Fluorescent Cell Linker Kit (Sigma) according to the manufacturer's protocol. After incubation with recipient cells that were pre-treated with exosomes labeled with PKH67. Cells were washed thrice with PBS, fixed with 4% paraformaldehyde for 30 min, and stained with DAPI for 20 min at RT. Finally, cells were observed under a confocal laser scanning microscope (Leica Microsystems AG).

### Co-culture assay

The co-culture assay was established using transwell membranes (pores 0.4 μm, Merck Millipore, USA) in a 12-well format. OS cells were pretreated with GW4869 (Selleck, USA; 20 μM) or DMSO for 24 h in advance. The co-culture was performed for 3-6 days whereas OS (5 × 10^4^ cells) on the permeable membranes were treated with GW4869 (20 μM) or DMSO, and then MRC5 cells (5 × 10^4^ cells at the beginning) below the membranes were ready for further cytological experiments.

### Animal study

For *in vivo* metastasis assays, each experimental group consisted of six 6-week-old male BALB/c-nu/nu mice. Briefly, 1.5 × 10^7^ cells with stably COL6A1 overexpressed or knockdown were suspended in 40 μL serum-free DMEM for each nude mouse (6 in each group), were injected intravenously through the tail vein into mouse. The mice were killed 8 weeks after injection. Tumor nodules formed on the lung surfaces were macroscopically determined and counted. The lungs were excised and embedded in paraffin. Our protocol was approved by the Committee on the Ethics of Animal Experiments of Sun Yat-sen University Cancer Center.

### Statistical analysis

Differences among the treatment groups were assessed via ANOVA using statistical software (SPSS, IBM, USA). The significance of differences between groups was estimated by Student's t-test, Wilcoxon test, as appropriate. The survival rates were calculated by the Kaplan-Meier, univariate and multivariate Cox proportional hazards model. Two-sided p-values were calculated, and a probability level of 0.05 was chosen for statistical significance. The data were expressed as mean ± SD of at least three separate experiments.

## Results

### COL6A1 is upregulated in human OS tissues and is related to poor outcomes

**Figure 1:**
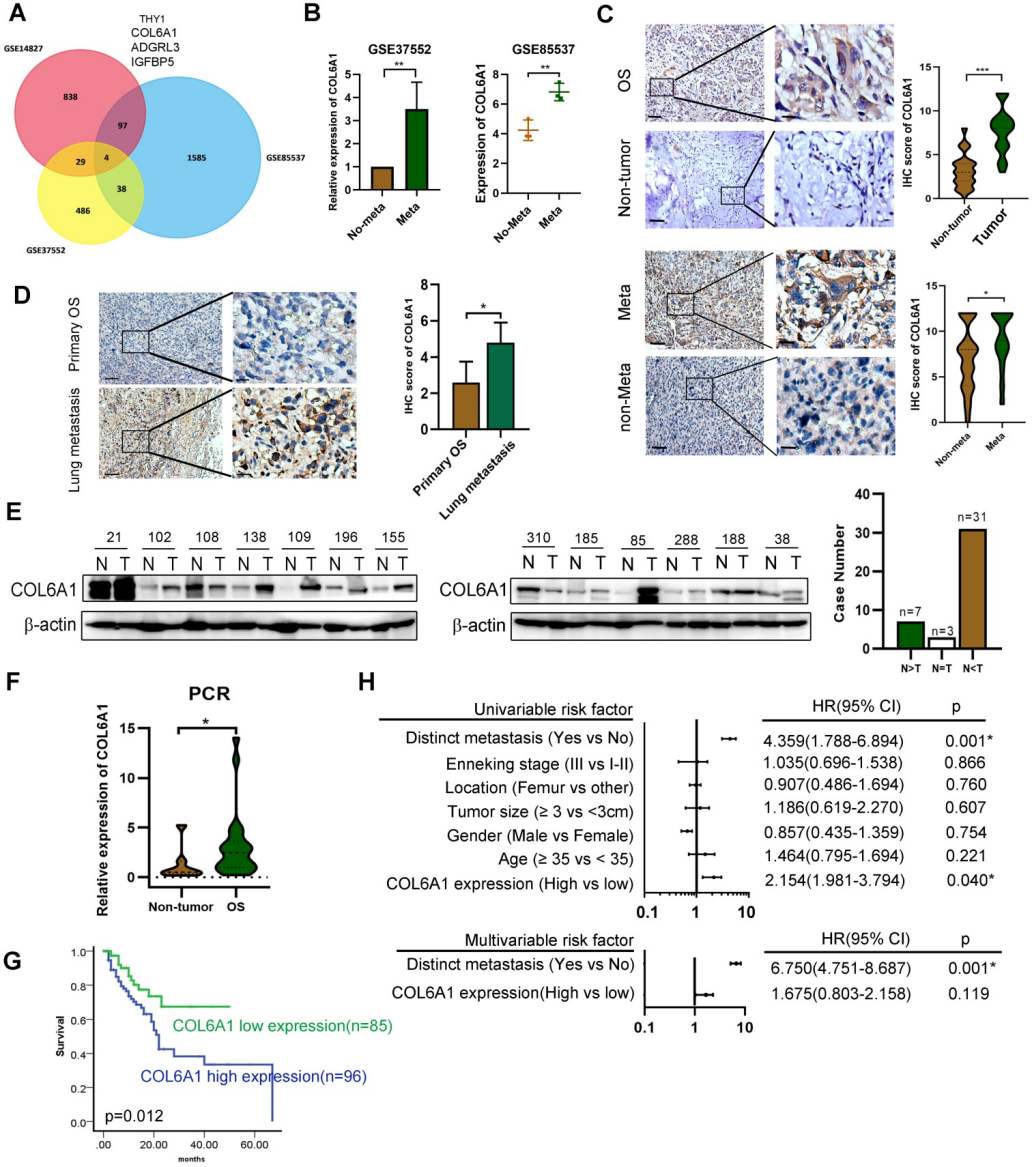
** Expression of COL6A1 in OS tissues with its clinical significance. A.** Venn diagram obtained from three microarray data: GSE14827 dataset (N = 27), the GSE85537 dataset (N = 6), and GSE37552 dataset (N = 4). **B.** Expression of COL6A1 in metastasis and non-metastasis OS tissues and cell lines. Data was explored from GSE37552 and GSE85537 database. **C**. Immunohistochemical staining of COL6A1 in primary OS samples and non-tumor tissues (n = 44), as well as OS tissues with metastasis (n = 29) and without metastasis (n = 152) (Scale bars: 200 µm, 50 µm). **D.** Immunohistochemical staining of COL6A1 in primary OS tissues (n = 181) and lung metastasis tissues (n = 9) (Scale bars: 200 µm, 50 µm).** E.** Expression of COL6A1 in 41 pairs of OS samples (T) and corresponding non-tumor tissues (N) was detected by Western blot analysis. **F.** QRT-PCR analysis of COL6A1 mRNA expression in OS samples (n = 22) and non-tumor tissues (n = 7). **G**. Comparison of overall survival of OS patients with high COL6A1 protein expression and low COL6A1 expression was detected by Kaplan-Meier curve. **H.** Forest plot showed the association between clinical parameters, COL6A1 expression and OS survival using univariate and multivariate analyses. (HR, hazard ratio; CI, confidence interval). *p < 0.05, **p < 0.01, ***p < 0.001 by Student's *t* test.

To identify genes that might predict OS metastasis, we analyzed three microarray datasets (GSE14827: N = 27, GSE85537: N = 6, GSE37552: N = 4) that included primary OS tissues, lung metastasis tissues, and OS primary and metastasis cell lines. Four of these genes (encoding THY1, COL6A1, ADGRL3, and IGFBP5) were present in all the three datasets (**Figure 1A**). As COL6A1 was shown to associate with tumor progression and metastasis in several cancer types, we selected it for further analysis. Moreover, the OS metastasis samples had significantly elevated COL6A1 expression (3.53-fold in GSE37552 and 1.56-fold in GSE85537), relative to non-metastasis samples (**Figure 1B**).

Expression of the COL6A1 protein in OS tissues (n = 181) was evaluated using immunohistochemistry, which revealed significantly higher expression in the OS tissues than in non-tumor tissues (n = 44) (**Figure 1C**). Furthermore, we found that COL6A1 expression was higher in metastatic OS cases (n = 29) than in non-metastatic OS cases (n = 152). The immunohistochemical scores were also higher in the lung metastasis tissues (n = 9) than in primary OS tissues (n = 181) (**Figure 1D**). Moreover, western blotting revealed significantly higher COL6A1 protein expression in the OS tissues than in case-matched non-tumor tissues (n = 41) (**Figure 1E**). We also performed qRT-PCR and found that the COL6A1 mRNA expression levels were higher in the OS tissues (n = 22) than in non-tumor tissues (n = 7). The clinical significance of COL6A1 expression was evaluated based on the clinicopathological characteristics of 181 patients with OS (**Table 1**). The results revealed that COL6A1 expression was significantly associated with larger tumor size, although COL6A1 expression was not significantly associated with age, sex, tumor location, or distinct metastasis. Clinical follow-up data revealed that high COL6A1 expression was strongly associated with poor overall survival (*p* = 0.012) (**Figure 1G**). Univariate and multivariate Cox regression analyses also confirmed that distinct metastasis was an independent predictor of survival among patients with OS (**Figure 1H**). Taken together, the results indicate that upregulation of COL6A1 was significantly associated with poor outcome in patients with OS.

### COL6A1 promotes *in vitro* and *in vivo* metastasis in OS models

**Figure 2:**
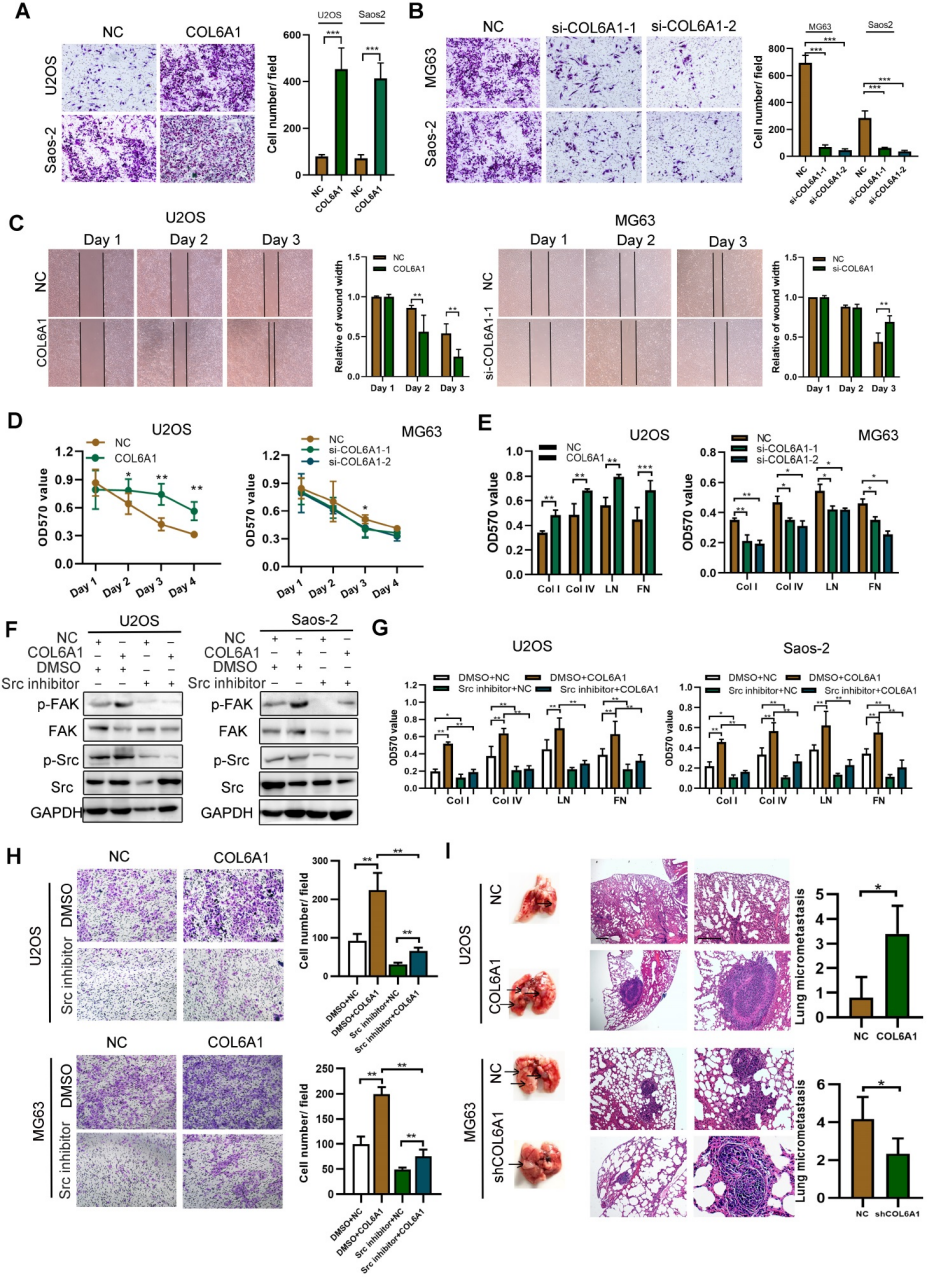
** COL6A1 promotes migration and invasion *in vivo* and *in vitro*. A.** Overexpression of COL6A1 increased OS cell migration and invasive abilities detected by transwell assay in OS cell lines, U2OS and Saos-2. **B.** Downregulation of COL6A1 by siRNA transfection resulted in a decrease in the migratory and invasive abilities of OS cells as determined by transwell analysis. **C.** The migration ability of OS cells was detected by wound-healing assay after COL6A1 or siCOL6A1 transfection. **D.** Anoikis assay: Cell viability determined by MTT in COL6A1 overexpressed, knockdown or control cells seeded at 10^5^ cells/well in a 96 poly-Hema culture plate.** E.** COL6A1-overexpressing, COL6A1 knockdown or control cells were subjected to cell-matrix adhesion assay to collagen I (Col I), collagen IV (Col IV), laminin (LN), and fibronectin (FN). **F**. Western blotting analysis of phosphorylation of FAK and Src and total FAK and Src in COL6A1-overexpressing and control cells upon treatment with vehicle or Src inhibitor (10 µM) for 24 h. **G.** COL6A1-overexpressing and control cells were subjected to cell-matrix adhesion assay to Col I, Col IV, and FN upon treatment with vehicle or Src inhibitor (10 µM) for 24 h. **H.** Cell migration potential was determined in COL6A1-overexpressing and control cells upon treatment with vehicle or Src inhibitor for 24 h according to transwell assays. **I.** Overexpression of COL6A1 increased the rate of lung metastasis after tail-vein injection of COL6A1 or control cells (n = 6 each group). Representative photographs of hematoxylin and eosin (H&E) staining in lung metastases tissues from mice orthotopically inoculated with COL6A1 and control cells (Scale bar: 200 µm, 50 µm). Data represent the mean ± SD of 3 separate determinations. *p < 0.05, **p < 0.01, ***p < 0.001 by Student's *t* test.

Further, we explored the functional role of COL6A1 in the tumorigenesis and/or progression in human OS. Relative to an osteoblast cell line (hFOB1.19), the OS cell lines (U2OS, Saos-2, and MG63) had higher COL6A1 mRNA and protein expression based on the qRT-PCR, western blotting, and immunofluorescence analyses (**Figure S1A**). The strongest COL6A1 expression was observed in the MG63 cell line, which also exhibited the highest metastatic potential based on the transwell assay than in other cells (**Figure S1B**). Next, the COL6A1 plasmid and siRNA transfection efficiency was confirmed by qRT-PCR and western blotting (**Figure S1C**). Without influencing cell proliferation, the expression of COL6A1 significantly increased colony formation, migration capability, and invasion capability, but decreased apoptosis in the OS cells (**Figure 2A-C and Figure S1D-G**). Anoikis is a special apoptotic process that is caused by a loss of or inappropriate cell adhesion, which leads to tumor recurrence and metastasis 21. We observed that COL6A1 transfection significantly promoted anoikis resistance under suspension culture conditions, and that *COL6A1*-knockdown cells were more sensitive to detachment-induced apoptosis than control cells (**Figure 2D**). Considering that COL6A1 is an important extracellular matrix protein, we used recombinant human COL6A1 (rhCOL6A1) to evaluate the effects of extracellular secreted COL6A1 on OS migration. However, COL6A1 protein expression and OS cell migration were not affected by rhCOL6A1 treatment (**Figure S1H-J**). These results indicate that intracellular COL6A1, rather than extracellular COL6A1, positively regulated OS cell migration, invasion, and resistance to anoikis.

We measured the OS cell-matrix adhesion and observed that *COL6A1* overexpression was associated with a significant increase in cell adhesion via collagen I, collagen IV, and fibronectin. In addition, *COL6A1* knockdown had the opposite effect (**Figure 2E and Figure S2A**), which indicated that COL6A1 facilitated OS cell-matrix adhesion. The focal adhesion kinase/Src kinase (FAK/Src) pathway regulates cell-matrix adhesion 22, which prompted us to evaluate the potential regulation of the FAK/Src pathway by COL6A1 in OS cells. We observed that *COL6A1* overexpression markedly increased the phosphorylation levels of FAK and Src, and that shRNA mediated *COL6A1* knockdown led to a significant decrease in FAK and Src phosphorylation (**Figure S2B**). Treatment of OS cells with a Src inhibitor significantly suppressed the effect of COL6A1 overexpression on Src and FAK activation, cell-matrix adhesion, and cell migration (**Figure 2F-H**). These results indicate that COL6A1 mediated OS cell-matrix adhesion and cell migration via increasing phosphorylation of Src and FAK. Cell lines with stable *COL6A1* overexpression or *COL6A1* knockdown were used to evaluate the *in vivo* effects of COL6A1 on metastasis using mouse xenograft models. **Figure 2I** shows that the COL6A1 group had a remarkably higher average number and size of metastatic pulmonary nodules relative to the control group. These results indicate that COL6A1 promotes OS cell invasion and migration *in vitro* and* in vivo*.

### p300/c-Jun induces COL6A1 upregulation by modulating H3K27 acetylation

**Figure 3:**
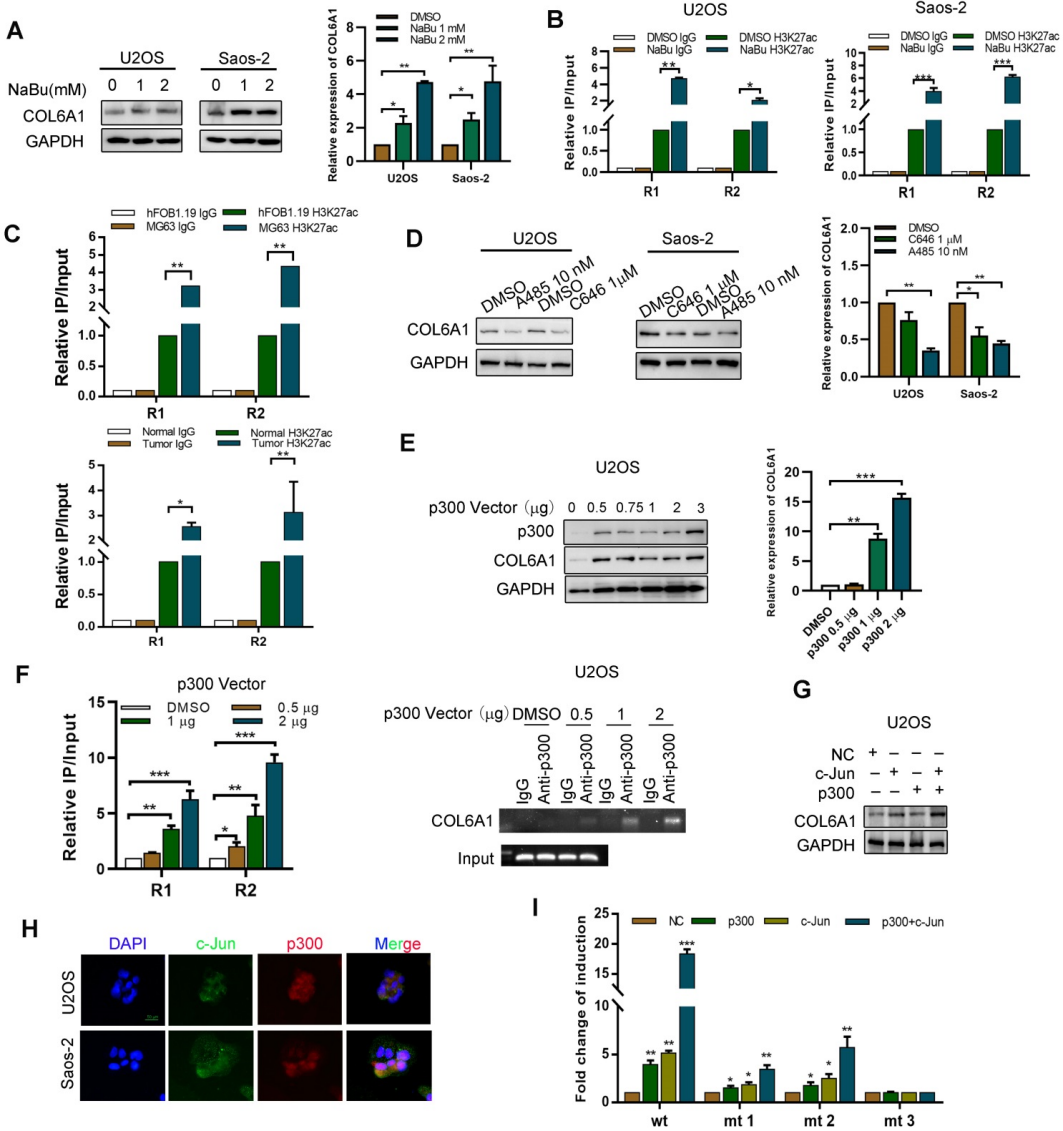
** P300/c-Jun induces an upregulation of COL6A1 by modulating H3K27 acetylation. A.** The expression of COL6A1 was detected by western blot and qRT-PCR upon treatment of different dose of NaBu in OS cell lines, U2OS and Saos-2. **B.** ChIP assay demonstrated that H3K27 acetylation occurred in the promoter of COL6A1 in OS cell lines using two primers upon treatment of NaBu. **C**. ChIP assay demonstrated that H3K27 acetylation occurred in the promoter of COL6A1 in OS cell lines MG63 and osteoblast cell line hFOB1.19, as well as case matched normal (bone) or OS tissues (n = 3) using two primers. **D.** The expression of COL6A1 was detected by western blot and qRT-PCR upon treatment of C646 or A485 in OS cell lines, U2OS and Saos-2. **E**. The expression of COL6A1 was detected by western blot and qRT-PCR upon p300 transfection in OS cell lines, U2OS and Saos-2. **F.** ChIP assay demonstrated that H3K27 acetylation occurred in the promoter of COL6A1 in OS cell lines U2OS upon p300 transfection (left panel). ChIP-PCR assay confirmed the interaction between p300 and COL6A1 promoter, and this interaction did not exist at the IgG promoter, an internal control (right panel). **G.** The expression of COL6A1 was detected after co-transfection of c-Jun and p300 plasmids in U2OS cell line. **H.** The co-localization of c-Jun and p300 was confirmed by confocal microscopy (Scale bars: 50 µm). **I.** U2OS cells were co-transfected with different combinations of wild type (wt) and c-Jun-1-site-mutated reporter constructs (mt 1, mt 2, and mt 3), p300, c-Jun, and control. The relative luciferase activity was analyzed as previously described. Data represent the mean ± SD of 3 separate determinations. *p < 0.05, **p < 0.01, ***p < 0.001 by Student's *t* test.

The potential mechanisms underlying COL6A1 overexpression in OS were evaluated using the UCSC Genome Bioinformatics Site (http://genome.ucsc.edu/). **Figure S3A** shows substantial enrichment of H3K27ac in the promoter region of *COL6A1*. We also found that expression of COL6A1 and H3K27ac enrichment levels were remarkably increased by treating OS cells with histone deacetylase inhibitor, sodium butyrate (NaBu) (**Figure 3A-B**). Moreover, we found greater enrichment of H3K27Ac at the *COL6A1* promoter in OS tissues and cells MG63 than in normal tissues and osteoblast cell hFOB1.19 (**Figure 3C**). These results suggest that histone acetylation/histone deacetylase-mediated histone modification may be an epigenetic mechanism that regulates COL6A1 transcription.

To evaluate whether histone acetyltransferase p300 plays a role in the activation of *COL6A1* gene transcription, we treated OS cells with two p300 inhibitors (C646 and A485). **Figure 3D** shows that this treatment decreased the COL6A1 mRNA and protein expressions. Furthermore, p300 overexpression in U2OS cells increased the expressions of COL6A1 mRNA and protein in a dose-dependent manner (**Figure 3E**). As expected, upregulation of p300 in OS cells increased the enrichment of H3K27ac at the *COL6A1* promoter, and p300 inhibition using A485 decreased the enrichment of H3K27ac (**Figure 3F and Figure SB**). As a transcriptional co-activator, p300 must be recruited to the target promoter by specific transcriptional factors, and the *COL6A1* promoter includes two c-Jun binding sites (-180 and-1970 bp). We found that c-Jun increases the expression and transcription activity of COL6A1 in a dose-dependent manner (**Figure S3C**). Western blotting revealed strong COL6A1 expression in cell co-transfected with p300 and c-Jun, which was significantly stronger than expression in cells transfected with p300 or c-Jun plasmids alone (**Figure 3G**). **Figure 3H** shows the nuclear co-localization of c-Jun and p300. To evaluate whether the two c-Jun-binding sites in the COL6A1 promoter contribute to the c-Jun-mediated activity of p300, we constructed three reporter plasmids with mutation at the -180-c-Jun site, -1970-c-Jun site, and both sites (**Figure S3D**). As shown in **Figure 3I,** relative to the wild type cells, all mutations reduced the synergistic action of p300 and c-Jun at the *COL6A1* promoter, and the double-mutated plasmid was associated with a significantly decreased synergistic action of p300 and c-Jun. These results indicate that binding of c-Jun with p300 promoted the enrichment of H3K27ac at the *COL6A1* promoter, which increased the transcription of COL6A1.

### COL6A1 promotes OS cell invasion and migration by suppressing STAT1 expression and activation

**Figure 4:**
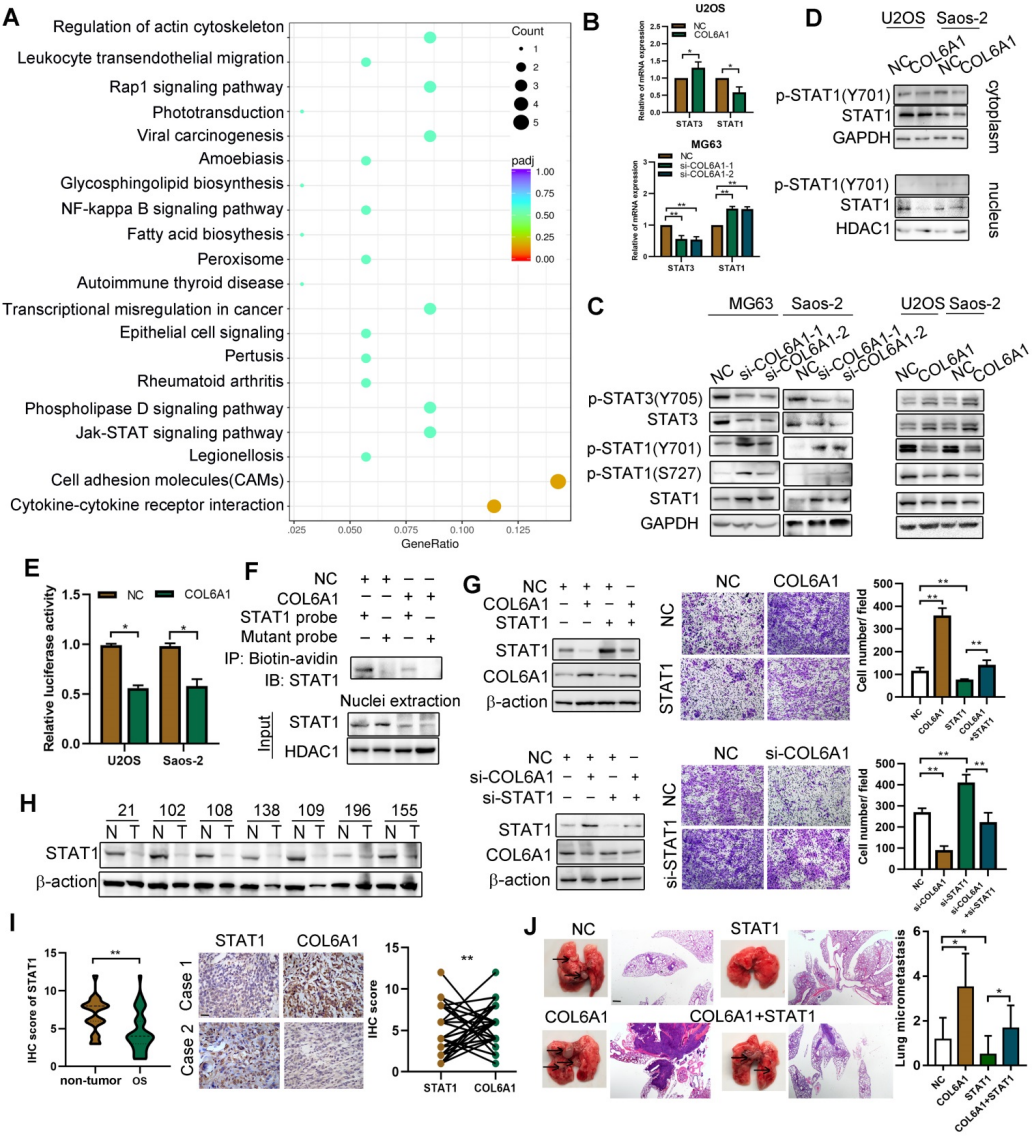
** COL6A1 promotes OS invasion and migration via suppressing STAT1 expression and activation. A.** Scatter plot of top 20 KEGG pathways enrichment of DEGs after COL6A1 transfection. Rich factor is the ratio of the DEG number to the background number in a certain pathway. The size of the dots represents the number of genes, and the color of the dots represents the range of the q-value. **B.** STAT1 and STAT3 mRNA levels were determined in OS cell with COL6A1 or siCOL6A1 transfection by qRT-PCR. **C.** Total and phosphorylated STAT1 and STAT3 protein expression levels were analyzed in COL6A1 overexpression and knockdown in OS cells by western blot. **D.** Nuclear and cytoplasmic STAT1 expression was analyzed in COL6A1 overexpression or control OS cells. **E.** STAT1 transcription luciferase reporter constructs were transiently transfected into the indicated cells, and luciferase activity was analyzed after 48 hours. **F.** Biotin pull-down assay with a STAT1 probe was used to determine its DNA binding after transfecting COL6A1. **G.** STAT1 overexpression decreased the migratory ability of COL6A1 overexpression OS cells. The migratory ability of OS cells was detected upon the indicated treatment (right panel). **H**. The expression of STAT1 in OS tissues was detected by western blot. **I.** Expression of STAT1 and COL6A1 in OS tissues was detected by immunohistochemistry and COL6A1 inversely correlated with STAT1 expression in human OS tissues (Scale bars: 100 µm). **J.** Overexpression of STAT1 decreased the rate of lung metastasis after tail-vein injection indicated cells (n = 6 each group). Representative photographs of H&E staining in lung metastases tissues from mice orthotopically inoculated with indicated treated cells (Scale bars: 400 µm). Data represent the mean ± SD of 3 separate determinations. *p < 0.05, **p < 0.01, ***p < 0.001 by Student's *t* test.

We performed RNA sequencing to identify genes that were differentially expressed between the *COL6A1*-overexpressing cells and control cells. A 2-fold screening filter was applied, which identified 71 upregulated genes and 136 downregulated genes in the cells with *COL6A1* overexpression. The most differentially expressed genes (DEG) are shown in **Figure S4A**; six DEGs were validated using qRT-PCR (**Figure S4B**). Analysis of gene ontology and the Kyoto Encyclopedia of Genes and Genomes revealed that the DEGs were related to cell adhesion molecules, Janus kinase (JAK)/ signal transducers and the activators of transcription (STAT) signaling pathway (**Figure 4A**). Considering that STAT1 and STAT3 are key regulators in the JAK-STAT pathway, we evaluated whether COL6A1 expression influenced STAT1 and STAT3 expression and activation in OS cells. **Figure 4B and C** show that STAT1 and p-STAT1 protein and mRNA levels are decreased after *COL6A1* transfection, while the STAT3 and p-STAT3 levels are increased. Previous research has indicated that STAT3 is a novel target for treating OS, although the role of STAT1 in OS remains unclear 23. Thus, we explored the effect of COL6A1 on STAT1 activity in OS. *COL6A1* overexpression decreased nuclear translocation, transcriptional activity and DNA binding of STAT1 based on the nuclear and cytoplasmic fraction assay, luciferase reporter and biotin pull down assay (**Figure 4D-F**). Moreover, we found that COL6A1 upregulated STAT3 expression and activation through inhibiting STAT1 expression (**Figure S4C-F**). These results indicate that COL6A1 suppresses STAT1 expression and activation in OS cells.

We confirmed the function of STAT1 in OS cells using gain-of-function and loss-of-function experiments. Relative to an empty vector, transfection of the *STAT1* vector resulted in significant increases in apoptosis and cell cycle arrest, as well as decreases in colony formation, cell migration, and cell invasion by suppressing epithelial-mesenchymal transition (EMT) in OS cells (**Figure S5A-I**). Rescue experiments were also used to determine whether the effect of COL6A1 on metastasis was mediated by repression of STAT1 in OS cells. The results revealed that the positive effects of *COL6A1* knockdown on cell migration were significantly impaired by STAT1 depletion. Moreover, transfection of *STAT1* significantly decreased the cell migration capability and EMT in cells with *COL6A1* overexpression (**Figure 4G**). Furthermore, we evaluated STAT1 protein expression by western blotting and observed higher expression in the non-tumor tissues than in OS tissues (**Figure 4H**). Based on immunohistochemistry, we observed that the expression of STAT1 and COL6A1 were inversely related (Fisher's exact test, p = 0.028) (**Figure 4I**). *In vivo*, the group with expression of STAT1 plus COL6A1 has less metastasis nodules than COL6A1 group, which suggested that elevated STAT1 expression suppressed the *in vivo* lung metastasis in *COL6A1*-overexpressing OS cells (**Figure 4J**). Taken together, these results indicate that COL6A1 promotes OS cell metastasis *in vitro* and *in vivo* by suppressing STAT1 expression and activation.

### COL6A1 interacts with E3 ligase SOCS5 to promote STAT1 degradation

**Figure 5:**
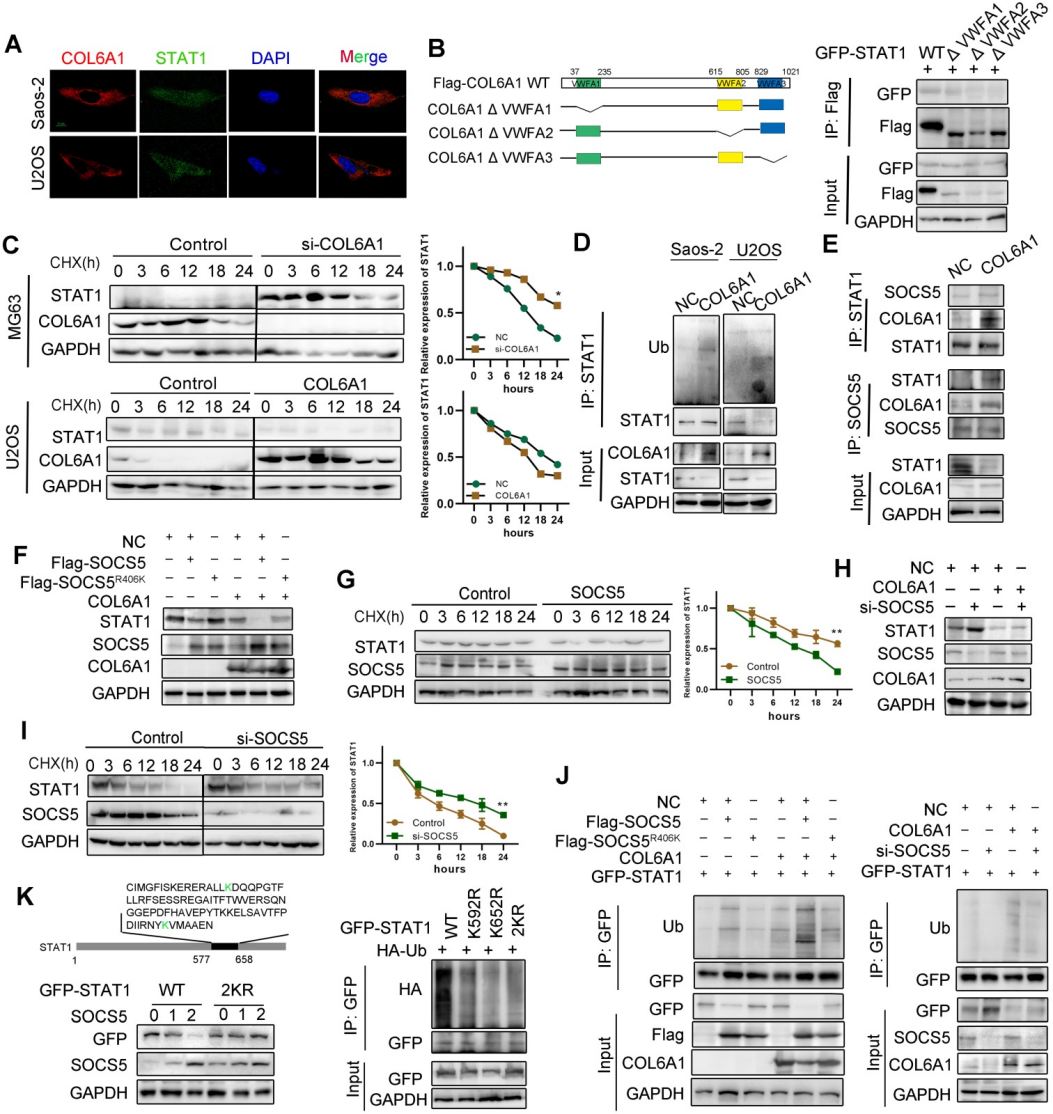
**COL6A1 interacted with E3 ligase SOCS5 to promote STAT1 degradation. A.** Co-localization of COL6A1 and STAT1 in ESCC cells were examined by using confocal microscopy (scale bar, 10 µm).** B.** The interaction of GFP-STAT1 and Flag-COL6A1 wide type, VWFA1, VWFA2, VWFA3 domains deletion mutations were detected by co-immunoprecipitation in 293T cells. **C.** COL6A1 was transfected into the OS cells in the presence of cycloheximide (CHX, 200 µg/mL) for indicated times. Cell lysates were immunoblotted by antibodies as indicated. The data were quantified using Image J software. **D.** STAT1 ubiquitination was detected by immunoprecipitation with anti-STAT1 antibody and immunoblotting with an anti-Ub antibody. **E.** The interaction of STAT1, COL6A1 and SOCS5 was detected by co-immunoprecipitation in U2OS. **F.** SOCS5 decreased STAT1 protein. U2OS cells were transfected with Flag-SOCS5 or Flag-SOCS5^R406K^ as well as control or COL6A1 transfection. The protein expression level of STAT1 was assayed by western blot. **G**. The cells expressing wide type SOCS5 were treated with CHX. The protein levels of STAT1 and SOCS5 were analyzed by western blot. **H**. Knockdown SOCS5 increased STAT1 protein. U2OS cells were transfected with si-SOCS5 as well as control or COL6A1 transfection. **I**. U2OS cells were transfected with control or SOCS5 siRNAs treated with CHX (200 µg/mL), the protein levels of STAT1 and SOCS5 were analyzed by western blot. **J.** SOCS5 ubiquitylates STAT1. U2OS cells were transfected with indicated plasmids or siRNA for 48 h. Cell lysates were immunoprecipitated with anti-GFP and analyzed by immunoblotting with indicated antibodies. **K**. U2OS cells were transfected with indicated plasmids. lysates were immunoprecipitated with anti-GFP, and western blots were performed to analyze the presence of indicated proteins and levels of ubiquitination. Data represent the mean ± SD of 3 separate determinations. *p < 0.05, **p < 0.01, ***p < 0.001 by Student's *t* test.

Next, we explored the mechanism by which COL6A1 regulated STAT1 activity in OS cells. Confocal microscopy experiments indicated co-localization COL6A1 and STAT1 in the Saos-2 and U2OS cell lines (**Figure 5A**). Co-immunoprecipitation revealed that STAT1 interacted with VWFA1 domain of COL6A1 (**Figure 5B and Figure S6A**). Ubiquitination and proteasomal degradation lead to STAT1 downregulation in several cancer types 20, and western blotting revealed dramatic dose-dependent increase in the STAT1 protein levels after MG132 treatment (**Figure S6B**). *COL6A1*-overexpressing cells had significantly shorter STAT1 protein half-lives, and the depletion of *COL6A1* expression restored STAT1 protein stability compared with control cells, as detected by cycloheximide chase assay (**Figure 5C**). These results supported that COL6A1 promoted proteasomal degradation of STAT1. We performed immunoprecipitation and western blotting and identified that COL6A1 expression was associated with increased STAT1 ubiquitination (**Figure 5D**). However, COL6A1 was pulled-down with and increased ubiquitination of the wild-type STAT1 and STAT1 mutated at two functionally active sites (Y701A and S727A), indicating that COL6A1-mediated degradation of STAT1 was independent of phosphorylation at Y701 or S727 (**Figure S6C-D**). COL6A1 increased the ubiquitination of STAT1 on the K63-linked ubiquitin chain, but not the K48-linked ubiquitin chain (**Figure S6E**).

We next investigated which E3 ubiquitin ligase mediated the protein degradation of STAT1 induced by COL6A1. Suppressor of cytokine signaling (SOCS5) is predicted with the highest confidence as a primary E3 ligase for STAT1 in UbiBrowser database (**Figure S6F**). We then confirmed a strong interaction between endogenous STAT1 and SOCS5 after COL6A1 transfection (**Figure 5E**). Moreover, deletion of the STAT1 SH2 domain abolished the interaction with SOCS5 (**Figure S6G**). To test whether SOCS5 affects the cellular level of STAT1, we overexpressed wild-type (wt) *SOCS5* in U2OS cells and found that the endogenous protein level of STAT1 was sharply reduced. However, ectopic expression of SOCS5^R406K^, which lacks ubiquitin ligase activity, did not affect the levels of STAT1, indicating that the E3 catalytic activity of SOCS5 was required for STAT1 protein destabilization (**Figure 5F**). Consistently, the half-life of STAT1 was significantly reduced in *SOCS5* overexpression cells (**Figure 5G**) but not in *SOCS5^R406K^* overexpression cells (**Figure S6H**). These results suggest that SOCS5 E3 ligase destabilizes STAT1 in OS cells. To investigate whether endogenous SOCS5 contributes to the COL6A1-induced protein degradation of STAT1, we transfected U2OS with *SOCS5* siRNA. Depletion of *SOCS5* resulted in a slight increase in the levels of STAT1, and the effect of SOCS5 was more substantial after COL6A1 transfection (**Figure 5H**). Furthermore, knockdown of *SOCS5* extended the half-life of STAT1, and the effect of SOCS5 was more significant after COL6A1 transfection (**Figure 5I and Figure S6I**). Consistent with these observations, overexpression of *SOCS5*, but not *SOCS5^R406K^,* significantly increased the ubiquitination of STAT1. Additionally, knockdown of *SOCS5* markedly reduced the ubiquitination of STAT1 (**Figure 5J**). To further understand the molecular basis of how STAT1 is ubiquitinated, we searched for specific sites in STAT1 isoforms that respond to SOCS5 E3 ligase and identified the K592 and K652 sites located in the SH2 domain of STAT1 to possibly affect STAT1 degradation. We generated series mutations in which the two K sites were mutated individually and in combination. As shown in ubiquitination assays, both individual and combination mutants of STAT1 were resistant to ubiquitination by SOCS5 (**Figure 5K**). Further, the half-lives of STAT1 2KR mutants were significantly extended in U2OS cells transfected with SOCS5 (**Figure S6J**). Taken together, these results demonstrated that COL6A1 interacted with E3 ligase SOCS5 to promote STAT1 degradation.

### Exosomal COL6A1 induces cancer-associated fibroblast activation to foster lung metastasis

**Figure 6:**
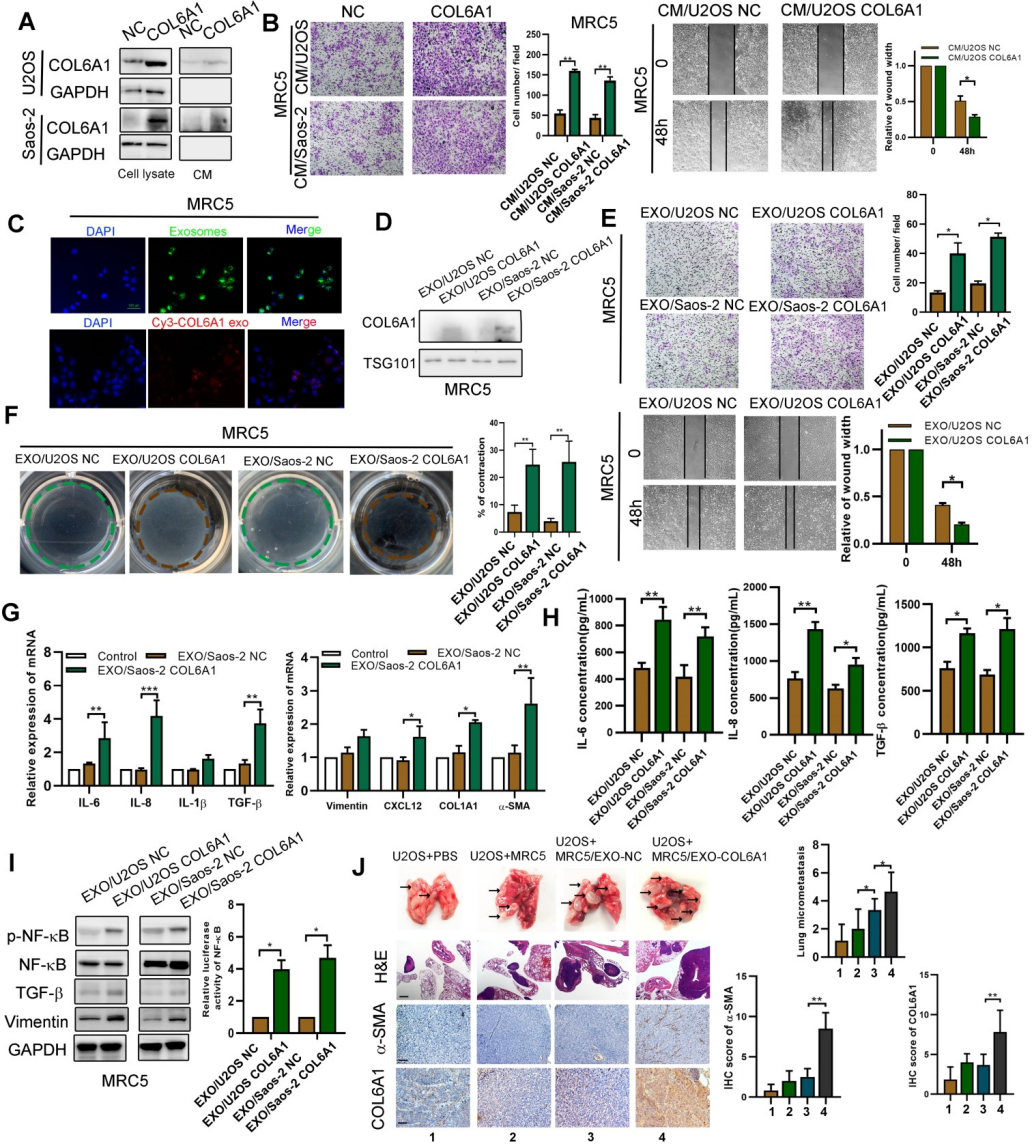
** Exosomes secreted from OS cells regulate fibroblasts activation to foster lung metastasis. A.** Examination of COL6A1 protein in the culture medium (CM) derived from COL6A1-overexpressing cells (CM/COL6A1) and control cells (CM/NC) by western blotting. **B**. Cell migration potential was determined in U2OS and Saos-2 cells incubated with CM/COL6A1 and CM/NC according to transwell assays. **C**. Confocal imaging showed the delivery of PKH67-labeled exosomes (green) to MRC5 cell (Scale bar, 100 µm). **D**. COL6A1 expression of MRC5 treated with exosomes released by indicated cells were detected by western blot. **E**. Migration ability of MRC5 treated with equal quantities of exosomes derived from indicated OS cells, detected by transwell assay and wound healing assay. **F.** MRC5 treated with exosomes released by indicated OS cells were assessed for their ability to contract collagen. Collagen contraction was quantified by the Image J software. **G.** Indicated gene expression of MRC5 treated with exosomes released by COL6A1-transfection OS cells or control were detected by qRT-PCR analysis. **H.** ELISA assay was performed to detect the expression of IL-6, IL-8 and TGF-β in MRC5 cells treated by indicated cells. **I.** NF-kB expression and transcription activation was detected by western blot and dual luciferase reporter assay in MRC5 cells treated by indicated cells. **J**. The rate of lung metastasis after tail-vein injection of indicated cells (n = 6 each group). Representative photographs of H&E staining (Scale bars: 400 µm) and COL6A1, a-SMA immunohistochemistry staining (Scale bars: 100 µm) in lung metastases tissues from mice orthotopically inoculated with indicated treated cells. Data represent the mean ± SD of 3 separate determinations. *p < 0.05, **p < 0.01, ***p < 0.001 by Student's *t* test.

We previously found that the expression of COL6A1 was positively correlated with α-smooth muscle actin (α-SMA), an activated CAF biomarker, in mouse lung metastasis tissues detected by IHC (data not shown). Thus, we chose MRC5 cells (human embryonic lung fibroblasts) as a model to explore whether tumor cell derived COL6A1 could promote OS metastasis by activating CAF*.* The COL6A1 protein was detectable in the culture medium (CM) from COL6A1-transfected cells and control cells, which suggested that both exogenous and endogenous COL6A1 can be secreted into the CM (**Figure 6A**). The wound healing assay and transwell assay results revealed that CM from the COL6A1-transfected cells (CM/COL6A1) significantly promoted the migration of MRC5 cells relative to CM from control cells (CM/NC) (**Figure 6B**). **Figure S7A** shows that MRC5 cells that were exposed to CM/COL6A1 had a higher expression of pro-inflammatory genes, such as IL-1β, IL-6, IL-8, and TGF-β, relative to MRC5 cells exposed to CM/NC. However, COL6A1 expression and MRC5 cell migration was not affected by rhCOL6A1 expression (**Figure S7B**). These results suggest that intracellular COL6A1, rather than extracellular COL6A1, promotes lung fibroblasts MRC5 cell migration.

Thus, we speculated that COL6A1 could be transferred from OS cells to MRC5 cells via exosomes, vital mediators between cancer cells and the stroma to transfer genetic messages. We purified and identified exosomes from the supernatant of OS cell cultures based on electron microscopy, nanoparticle tracking analysis, and immunostaining (**Figure S7C**). Furthermore, increased COL6A1 protein expression and cell migration ability was observed in the recipient MRC5 cells after treatment using U2OS CM/COL6A1, although an exosome blocker (GW4869) abolished this effect (**Figure S7D**). These results indicate that the unique COL6A1 content of OS-derived exosomes functions as a migratory regulator in MRC5 cells. The exosome tracing assay showed green (PKH67-labelled OS cell-derived exosomes) and red (Cy3-labeled, COL6A1 plasmid-transfected OS cell-derived exosomes) spots in the recipient MRC5 cells after co-culturing with OS cells (**Figure 6C**). The expression of COL6A1 was also increased in MRC5 cells following treatment with exosomes derived from COL6A1-expressing tumor cells (EXO/COL6A1) (**Figure 6D**). Transwell and wound healing assays revealed that a greater number of fibroblasts migrated in the EXO/COL6A1 group than in the EXO/NC group (**Figure 6E**). Moreover, activated CAFs can enhance matrix adhesions, which results in increased contraction of the collagen gel matrices 12. Therefore, we assessed the effects of OS cell-derived exosomes on fibroblast-mediated collagen contraction, which revealed markedly enhanced contraction abilities after treatment with EXO/COL6A1 (vs. EXO/NC, **Figure 6F**). The MRC5 cells exposed to EXO/COL6A1 showed significantly higher expression of pro-inflammatory genes and enhanced secretion of IL-6, IL-8, and TGF-β based on the qRT-PCR and ELISA results (**Figure 6G-H and Figure S7E**). As IL-1b, IL-6, and IL-8 are well-known targets of nuclear factor-kB (NF-kB), we evaluated NF-kB signaling in the experimental models. The MRC5 cells that had been exposed to CM/COL6A1 had increased NF-kB expression and activation (vs. the CM/NC group, **Figure 6I**), and similar results were obtained when the MRC5 cells were transfected with COL6A1 or control vectors (**Figure S8A-F**).

Intravenous administration of exosomes from COL6A1-transfected OS cells markedly contributed to the *in vivo* formation of lung metastasis and CAF enrichment based on the immunohistochemical evaluation of α-SMA expression (**Figure 6J**). We then evaluated serum-derived exosomes from OS patients and healthy controls, and revealed that OS patients with lung metastasis from OS had higher serum exosome concentrations and serum-based exosomal expression of COL6A1 than non-metastasis OS patients and the healthy controls (**Figure S8G-H**). These results suggest that exosomes derived from *COL6A1*-overexpressing OS cells contributed to fibroblast activation and led to lung metastasis.

### Activated fibroblasts promote OS metastasis by mediating TGF-β/COL6A1 signaling pathway

Tumor progression is mediated by CAFs secreting multiple pro-inflammatory cytokines and chemokines 24. Thus, we transfected the COL6A1 or control vectors into MRC5 cells and co-cultured them with the OS cell lines (U2OS and Saos-2).**Figure S9A-C** shows that OS cells co-cultured with *COL6A1*-overexpressing MRC5 cells (MRC5 COL6A1) exhibited enhanced cell motility, a higher proportion of CD133+ cells, and greater EMT progression, relative to the control cells (MRC5 NC). However, a TGF-β inhibitor (LY2109761) partially reversed these effects in OS cells, which suggests that COL6A1-transfected activated fibroblasts promoted OS cell stemness, EMT, and cell invasion by secreting TGF-β.

**Figure 7:**
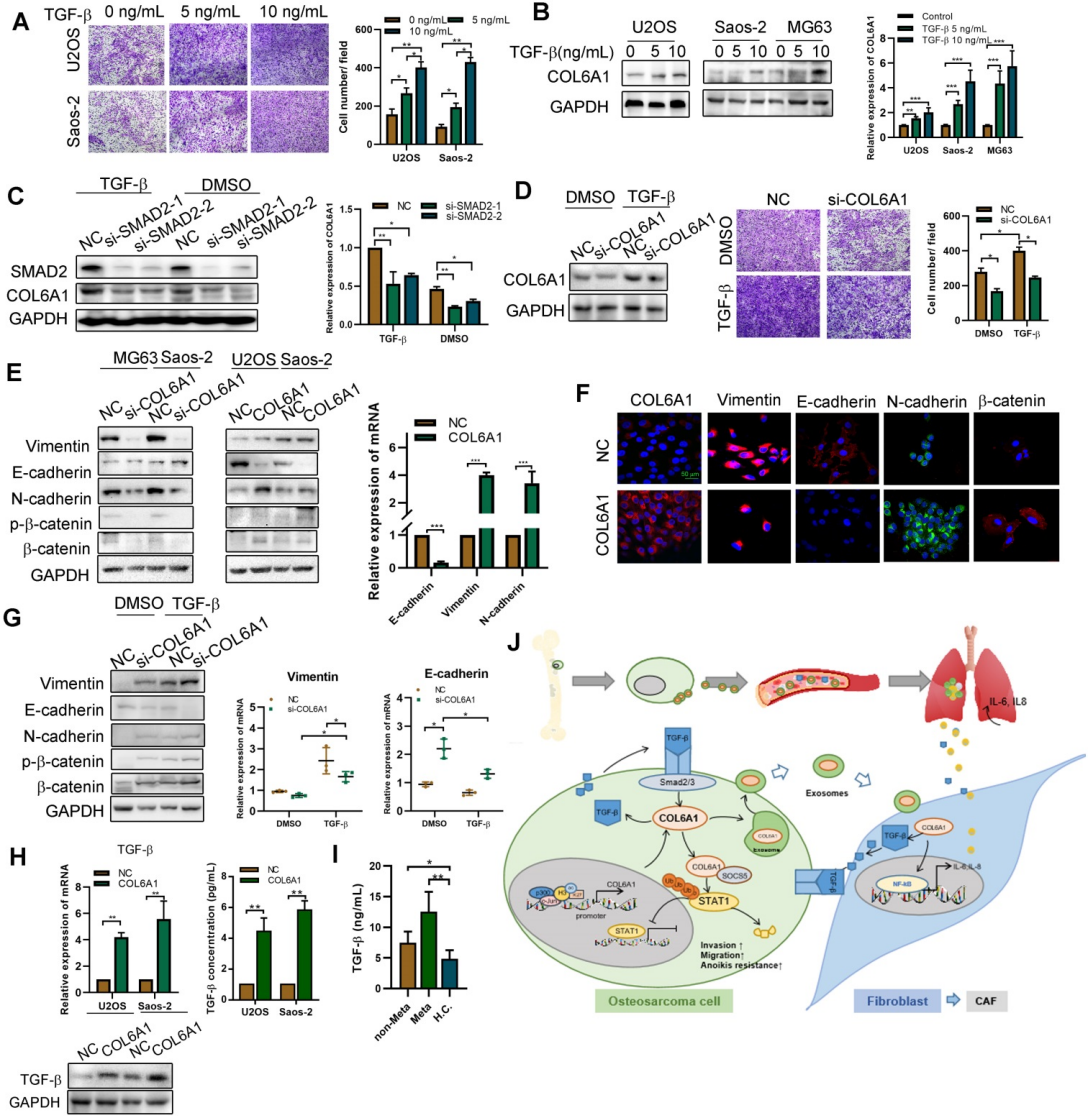
** Activated fibroblasts promote OS metastasis by secreting TGF-βA.** The cell invasion ability was detected by transwell assay after TGF-β treatment. **B.** COL6A1 expression was detected by western blot, confocal microscope and qRT-PCR in OS cells exposed to mock vehicle or TGF-β (0-10 ng/mL) for 12 h.** C**. COL6A1 protein and mRNA expression in U2OS cells transfected with siRNAs targeting SMAD2 exposed to mock vehicle or TGF-β (5 ng/mL) for 12 h. **D.** COL6A1-knockdown Saos-2 cells were treated with TGF-β, western blot were performed to detect the expression of COL6A1. **E**. COL6A1-knockdown Saos-2 cells were treated with TGF-β and transwell assay were performed to detect the migration ability of OS cells.** F.** The expressions of E-cadherin, N-cadherin, β-catenin and vimentin in OS cells with COL6A1 or si-COL6A1 transfection by western blot, qRT-PCR and confocal microscope (Scale bars: 50 µm). **H.** COL6A1-knockdown U2OS cells were treated with TGF-β, western blots and qRT-PCR were performed on cell lysates with the indicated antibodies**.** The protein and mRNA expression of TGF-β was detected in COL6A1 overexpressed OS cells. **I.** The concentration of TGF-β in OS patients' serum was detected by ELISA (non-Meta: non-metastasis OS patients; Meta: lung metastasis OS patients; H.C.: healthy control). **J.** Schematic diagram summarizing how COL6A1 promotes OS metastasis via suppressing STAT1 expression and activating CAFs. Exo, exosome. Data represent the mean ± SD of 3 separate determinations. *p < 0.05, **p < 0.01, ***p < 0.001 by Student's *t* test.

As TGF-β signaling pathway plays an important role in EMT and tumor metastasis, we found that TGF-β increased the migration ability of OS cells in a dose-dependent manner and also profoundly induced COL6A1 mRNA and protein expression in OS cell lines (**Figure 7A-B and Figure S9D**). To confirm that TGF-β induced COL6A1 expression through SMAD complex (a major regulator of TGF-β signaling), we depleted SMAD2 levels by siRNA transfection in Saos-2 and U2OS cells. **Figure 7C** shows that *SMAD2* knockdown abolished the TGF-β-induced COL6A1 expression at the mRNA and protein levels in both the cell lines. Moreover, silencing of *COL6A1* expression suppressed the OS cell migration ability that was induced by TGF-β (**Figure 7D**). These results indicate that TGF-β may be a critical regulator of COL6A1 expression in OS cells. Further, we measured the expression of EMT-related factors in Saos-2 and U2OS cells and found that knockdown of COL6A1 significantly increased the protein expression of E-cadherin and reduced expression of N-cadherin, vimentin and β-catenin; whereas, overexpression of COL6A1 rescued these expression changes (**Figure 7E-F**). We examined the effects of *COL6A1* silencing on TGF-β-induced EMT markers; the analysis indicated that *COL6A1* silencing significantly increased the TGF-β-induced inhibition of E-cadherin mRNA and protein expression in U2OS cells (**Figure 7G**). Immunofluorescence further confirmed that ectopic expression of *COL6A1* potentiated TGF-β-induced changes in EMT marker expression (**Figure S9E**). A previous study indicated that COL6A1 promotes tumor metastasis partially via enhanced TGF-β signaling; we also observed that COL6A1 expression increased the expression of TGF-β based on the results from western blotting, qRT-PCR, and ELISA (**Figure 7H**). By ELISA, serum samples from patients with OS had higher TGF-β concentrations than samples from healthy controls. Moreover, patients with lung metastasis from OS had higher serum TGF-β concentrations than patients with non-metastatic OS (**Figure 7I**). These results indicate that COL6A1 plays an important role in TGF-β-induced EMT and highlights a positive feedback loop that activates COL6A1 and TGF-β in OS.

## Discussion

To the best of our knowledge, this is the first study to demonstrate that COL6A1 strongly modulates the metastatic phenotypes of OS cells *in vitro* and *in vivo*. The mechanisms include suppression of STAT1 expression and activation by promoting its ubiquitination and proteasomal degradation in OS cells. Furthermore, COL6A1 was packaged in exosomes and helped activate CAFs to promote OS metastasis (**Figure 7J**). We believe that this is the first study to evaluate that COL6A1 acts on intracellular signaling pathway and extracellular fibroblasts to promote OS metastasis.

Previous studies have indicated that COL6A1 is upregulated in several malignancies, including glioblastoma and astrocytoma 25, 26, although they failed to investigate the underlying mechanism(s). The present study revealed that H3K27ac was enriched at the COL6A1 promoter in OS cells. This is important because histone acetylation and deacetylation are elaborately controlled and highly specific regulatory processes that lead to activation and repression, respectively, of some cancer-related genes 27. For example, histone deacetylation around the NOTCH1 promoter inhibits its protein expression in small cell lung carcinoma 28. The p300/CBP histone acetyltransferase can also be recruited at the PD-L1 promoter, which leads to increased PD-L1 expression in breast cancer 29. The results indicate that the c-Jun transcription factor functions synergistically with p300 to activate the COL6A1 promoter, suggesting that histone acetylation modification plays an important role in regulating the transcription and expression of COL6A1.

Under physiological conditions, COL6A1 exerts different cytoprotective functions, including inhibition of apoptosis, regulation of cell differentiation, and promotion of cell growth 4. However, other studies have indicated that COL6A1 can promote metastasis as knockdown of COL6A1 suppressed the metastatic ability of lung cancer and pancreatic cancer by regulating expression of proteins related to EMT 25.The present study indicated that increased expression of COL6A1 in OS cells significantly promoted cell adhesion, migration and invasion *in vitro* and *in vivo*. The gain- and loss-of-function experiments also provided strong evidence that COL6A1 promoted OS cell adhesion to extracellular matrix components (collagen I, collagen IV, and fibronectin) and increased the phosphorylation levels of FAK and Src, which is consistent with the KEGG analysis of RNA-seq results that COL6A1 was closely related with cell adhesion molecular pathway. Furthermore, we found that COL6A1 enhanced anoikis resistance, which is a prerequisite for tumor metastasis. Taken together, these results indicate that COL6A1 plays important roles in OS cell invasion by promoting cell attachment to the extracellular matrix and cell survival after detachment from extracellular matrix.

We also explored the downstream molecular mechanisms by which COL6A1 induced metastasis in the OS cells. We determined that the JAK/STAT pathway was involved and that STAT1 was one putative target. The experiments revealed that STAT1 plays a tumor suppressor role in OS, which is consistent with previous reports that showed that the forced expression of STAT1 can repress tumor metastasis in variety of cancer types, such as fibrosarcoma, head and neck squamous cell carcinoma, gastric cancer, breast cancer, and lung adenocarcinoma 30. We observed that the reintroduction of STAT1 in COL6A1-overexpressing OS cells antagonized the effects of COL6A1 in OS cell invasion and migration. Previous study indicated that proteasomal degradation led to low expression of STAT1 in cancer 31, consistent with the data that proteasome inhibitors can prolong the activation and increase total expression of STAT1. Moreover, the ubiquitination and degradation of STAT1 could be mediated by several proteins, including E3 ligase, ERK, osteopontin, and SMAD ubiquitination-regulatory factor 1/Smurf1, in human breast cancer cells and murine mammary epithelial tumor cells 20, 30, 32. The present study revealed that the E3 ligase SOCS5 could bind with COL6A1 to promote the degradation of STAT1 via ubiquitination, which regulated its expression in OS cells. These results suggest that the COL6A1/STAT1 axis plays an important role in OS invasion and migration.

CAFs enhance tumor invasion via specific communications with cancer cells 33. Thus, based on our previous study, we speculated that COL6A1, as an extracellular protein, could have significant effects on the tumor-CAF interaction. We observed that COL6A1 exists in the OS cell culture medium and promotes the migration of lung fibroblasts (MRC5 cells) and increases the expression of α-smooth muscle actin, IL-6, and IL-8, which are biomarkers for CAF activation 33. However, rhCOL6A1 had no effect on the migration of MRC5 or OS cells, which suggested that COL6A1 could be internalized through some carriers to intracellularly drive the activation of CAFs. Exosomes are also important mediators of cell interaction by which tumor-derived exosomes can drive the activation of normal fibroblasts and differentiation into CAFs, as observed in colon cancer and ovarian cancer models 24, 34. For example, liver tumor-derived exosomal miR-1247-3p could convert fibroblasts to CAFs in the lung pre-metastatic niche 19. The present study indicated that COL6A1 was present in OS cell-derived exosomes and helped drive the conversion of fibroblasts into CAFs. As the most abundant cells in the tumor stroma, CAFs enhance the metastasis of the head, neck, and oral cancers 35. However, it remains unclear how CAFs participate in cancer cells acquiring an aggressive phenotype, as well as the molecular mediators that are involved in these processes. Ren et al. reported that CAF-mediated increased transcription of HOTAIR in breast cancer cells promoted invasion and metastasis via the secretion of TGF-β, which is a crucial mediator of the cross-talk between stromal and cancer cells within the tumor microenvironment 36. The present study also revealed that activated CAFs enhanced the invasive potential of OS cells by secreting TGF-β, which induced COL6A1 expression and the EMT, and ultimately facilitated OS metastasis. All results suggest that exosomal COL6A1 in the extracellular environment may serve as a significant factor that promotes OS metastasis via tumor cell-fibroblast interactions.

The present study has a few limitations. First, although we detected exosomal COL6A1 expression in the serum samples of OS patients, more cases would be needed to strengthen the findings. Second, how OS cell-derived exosomes were internalized by OS cells and fibroblasts, or COL6A1 was packaged into exosomes remained to be studied. Third, the expression of COL6A1 in CAFs and normal fibroblasts from patients with OS was not evaluated.

## Conclusion

Our results indicated that COL6A1 was upregulated in OS tissues and related with lung metastasis and poor survival of OS patients. The transcription factor c-Jun bound p300 increased the enrichment of H3K27ac at the promoter region of COL6A1, thus resulting in the upregulation of COL6A1 in OS. Furthermore, COL6A1 promoted OS metastasis by suppressing STAT1 via promoting its ubiquitination proteasome degradation. Moreover, COL6A1 can be packaged as OS cell-derived exosomes, activating CAFs to promote OS metastasis. The activated CAFs could promote OS cell invasion and migration by secreting TGF-β. In conclusion, we demonstrated that COL6A1 promotes OS metastasis via suppressing STAT1 expression and activating CAF. Our study elucidates a new molecular mechanism underlying OS cells metastasis to lung and the crosstalk between tumor cells and fibroblasts, which contributes to efficient prevention and therapeutic strategies for OS.

## Supplementary Material

Supplementary figures and tables.

## Figures and Tables

**Table 1:** The correlation between COL6A1 expression and clinical pathology parameters in OS

Parameters	Case (n = 181)	Expression		Result (*p-*value)
		High (n = 96)	Low (n = 85)	
**Age**				
≥24	80	45	35	
<24	101	51	50	0.457
**Gender**				
Male	98	48	50	
Female	83	48	35	0.259
**Tumor site**				
Femur	59	34	25	
Tibia	43	21	22	
Jawbone	26	13	13	
Other	55	28	25	0.85
**Tumor size**				
>3 cm	61	40	21	
<3 cm	120	56	64	0.018*
**Enneking stage**				
I	39	24	15	
II	46	19	27	
III	96	53	43	0.145
**Distinct metastasis**				
No	152	76	76	
Yes	29	20	9	0.069

## Abbreviations

COL6A1: Collagen type VI alpha 1
CAFs: Cancer Associated Fibroblasts
CSCs: Cancer Stem Cells
ChIP: Chromatin Immunoprecipitation
CHX: Cycloheximide
DEGs: Differentially Expressed Genes
DMEM: Dulbecco's Modified Eagle's Medium
ECM: Extracellular matrix
EMT: Epithelial-Mesenchymal Transition
ELISA: Enzyme-Linked Immunosorbent Assay
FBS: Fetal Bovine Serum
FAK/Src: Focal Adhesion Kinase/Src kinase
GO: Gene ontology
HAT: Histone Acetylation
HDAC: Aistone Deacetylase
IHC: Immunohistochemistry
IL: Interleukin
JAK: Janus Kinase
KEGG: Kyoto Encyclopedia of Genes and Genomes
MiRNA: microRNAs
MTT: 3-(4,5-dimethyl-2-thiazolyl)-2,5-diphenyl-2H-tetrazolium bromide
NF-κB: Nuclear Factor-κB
NC: Negative Control
Ub: Ubiqution
OS: Osteosarcoma
rhCOL6A1: Recombination Human COL6A1
SOCS5: Suppressor of Cytokine Signaling 5
STAT: Signal Transducers and Activators of Transcription
TME: Tumor Microenvironment
TGF-β: Transforming Growth Factor-β
WT: Wild type
