# H3K27 acetylation activated-COL6A1 promotes osteosarcoma lung metastasis by repressing STAT1 and activating pulmonary cancer-associated fibroblasts: Erratum

**DOI:** 10.7150/thno.74233

**Published:** 2022-05-28

**Authors:** Ying Zhang, Zhaoyong Liu, Xia Yang, Weiqing Lu, Yelong Chen, Youbin Lin, Jin Wang, Suxia Lin, Jing-Ping Yun

**Affiliations:** 1 Sun Yat-sen University Cancer Center; State Key Laboratory of Oncology in South China; Collaborative Innovation Center for Cancer Medicine, Guangzhou 510060, China.; 2 Department of Pathology, Sun Yat-sen University Cancer Center, Guangzhou 510060, China.; 3 Department of Orthopedics, First Affiliated Hospital of Shantou University Medical College, No.57 Changping Road, Shantou, Guangdong 515041, China.; 4 Department of Orthopedics, Sun Yat-sen University Cancer Center, Guangzhou 510060, China.

The authors regret that the original version of our paper unfortunately contained some incorrect representative images. The transwell images in Figure 2A, Figure 2B and Figure 4G had been misused during figure assembly. The correct version of the Figure 2A, Figure 2B and Figure 4G appears below.

The authors confirm that the corrections made in this erratum do not affect the original conclusions. The authors apologize for any inconvenience that the errors may have caused.

## Figures and Tables

**Figure 2:**
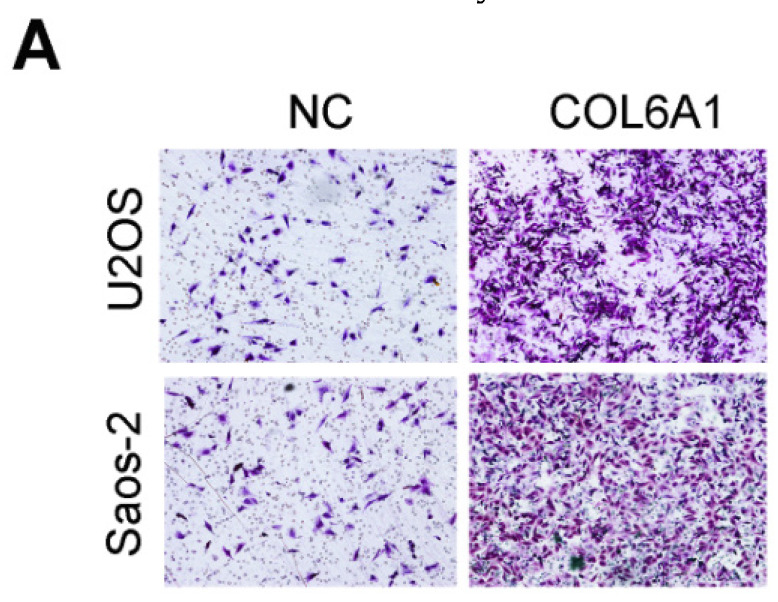

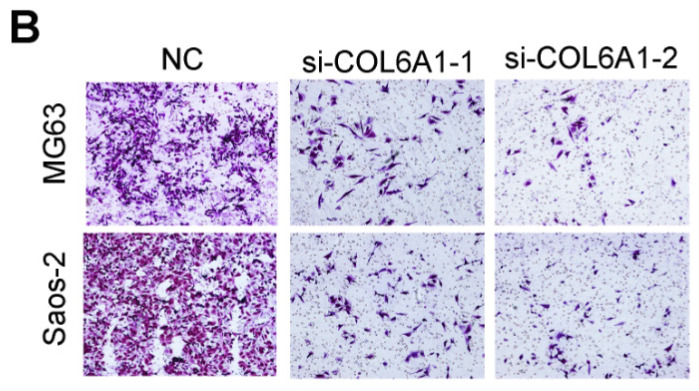
** Corrected figures. A**. Overexpression of COL6A1 increased OS cell migration and invasive abilities detected by transwell assay in OS cell lines, U2OS and Saos-2. **B.** Downregulation of COL6A1 by siRNA transfection resulted in a decrease in the migratory and invasive abilities of OS cells as determined by transwell analysis.

**Figure 4:**
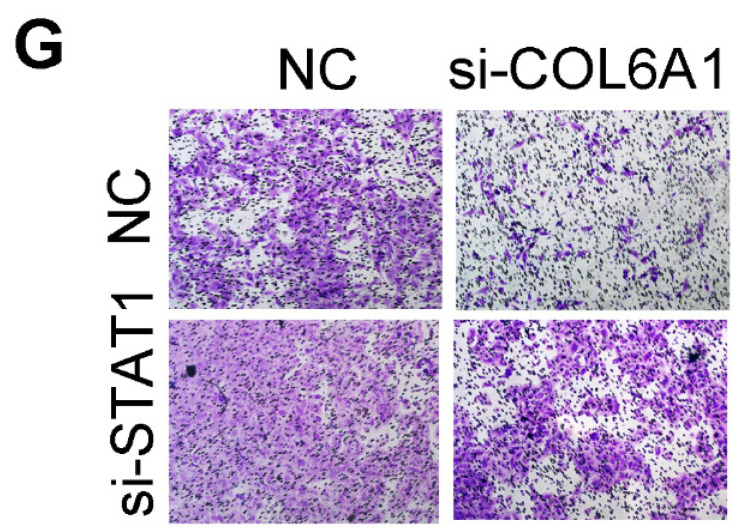
** Corrected figure. G.** STAT1 overexpression decreased the migratory ability of COL6A1 overexpression OS cells. The migratory ability of OS cells was detected upon the indicated treatment (right panel).

