# Supplementary material for: H3K27 acetylation activated-COL6A1 promotes osteosarcoma lung metastasis by repressing STAT1 and activating pulmonary cancer-associated fibroblasts
Source: Theranostics. 2021 Jan 1;11(3):1473–92. doi: 10.7150/thno.51245 (PMC7738898; doi:10.7150/thno.51245)
Supplement: Supplementary file 1 — Supplementary figures and tables. [file thnov11p1473s1.pdf]

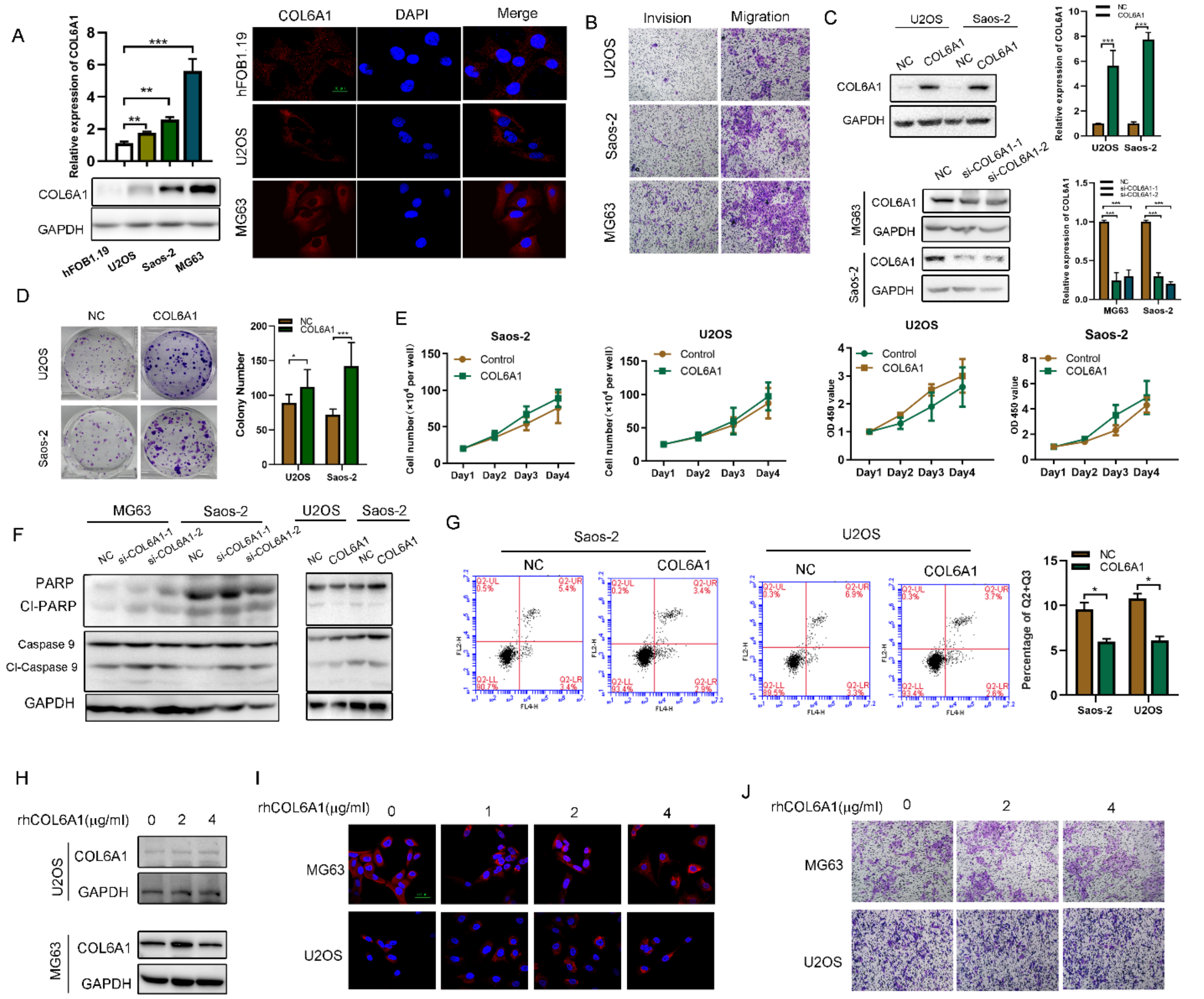

Figure S1. **The function of COL6A1 in OS cells**. **A**. The expression of COL6A1 in OS cell lines U2OS, Saos-2 and MG63 and osteoblast cell lines hFOB1.19 was detected by qRT-PCR, westernblot and Immunofluorescence. Confocal imaging showed that COL6A1 located in the cytoplasm (Scale bars: 50 μm). **B.** The invasion ability of OS cell lines U2OS, Saos-2 and MG63 was detected by transwell assay. **C**. The transfection efficiency of COL6A1 plasmid and siRNA was confirmed by Western blot and qRT-PCR analysis. **D**. Colony formation assay was performed on control and COL6A1-overexpressing Saos-2 and U2OS cells. **E**. A cell counting and CCK8 was performed on control and COL6A1-overexpressing OS cells. **F**. Westernblot was performed to detect the apoptosis on COL6A1-overexpressing and knockdown OS cell lines. **G**. Annexin V-PI assay was performed to detect the apoptosis on COL6A1-overexpressing OS cells. **H.** The effect of rhCOL6A1 on the expression of COL6A1 in OS cells detected by westernblot. **I.** The effect of rhCOL6A1 on the expression of COL6A1 in OS cells detected by immunofluorescence (Scale bars: 50 μm). **J.** The effect

of rhCOL6A1 on the migration of OS cells detected by transwell assay. Data represent the mean ± SD of 3 separate determinations. *p < 0.05, **p < 0.01, ***p < 0.001 by Student's t test.

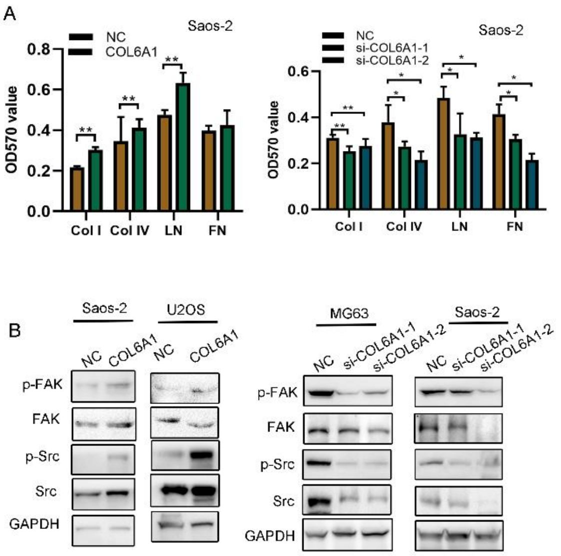

Figure S2. **The COL6A1 effects on cell adhesion signaling pathway. A** COL6A1-overexpressing, COL6A1 knockdown or control cells were subjected to cell-matrix adhesion assay to collagen I (Col I), collagen IV (Col IV), laminin (LN), and fibronectin (FN). **B.** Western blotting analysis of phosphorylation of FAK and Src and total FAK and Src in COL6A1-overexpressing, COL6A1 knockdown and control cells. Data rep resent the mean ± SD of 3 separate determinations. *p < 0.05, **p < 0.01, ***p < 0.001 by Student's t test.

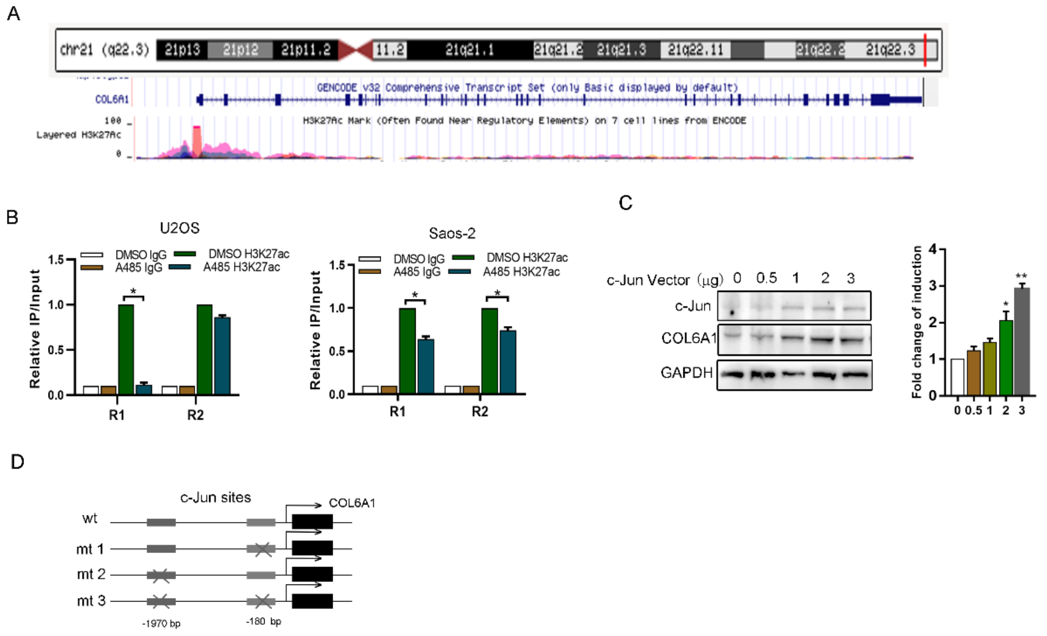

Figure S3. **H3K27 acetylation in COL6A1 promoter region. A**. The genome bioinformatics analysis showed that H3K27ac was enriched at the promoter of COL6A1 by using UCSC Genome Bioinformatics Site (http://genome.ucsc.edu/). **B.** ChIP assay demonstrated that H3K27 acetylation occurred in the promoter of COL6A1 in OS cell lines, U2OS and Saos-2, using two primers upon treatment of A485. **C.** The expression and transcription activation of COL6A1 was detected by westernblot and luciferase reporter upon transfection of c-Jun in U2OS. **D**. Schematic presentation of c-Jun binding sites in the promoter region of COL6A1. Data represent the mean ± SD of 3 separate determinations. *p < 0.05, **p < 0.01, ***p < 0.001 by Student's t test.

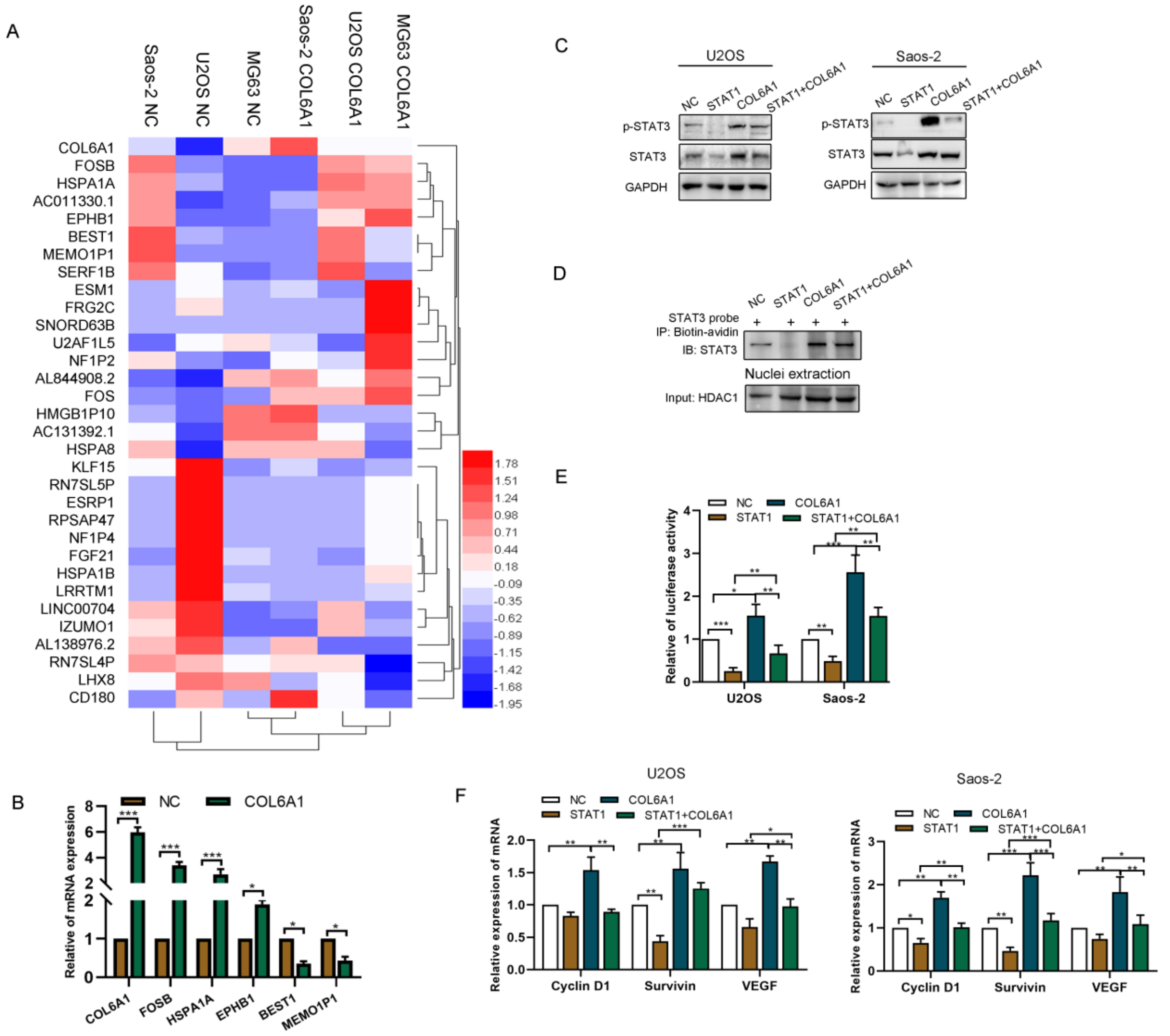

Figure S4. A. Top different expression genes in COL6A1 overexpression and control cell using three OS cell lines was shown. **B.** The mRNA expression levels of the indicated genes were determined in COL6A1 overexpression and control cell lines using qRT-PCR. **C.** The expression of p-STAT3 and STAT3 was detected in indicated transfected OS cells. **D**. Biotin pull-down assay with a STAT3 probe was used to determine its DNA binding after transfecting COL6A1. **E.** STAT3 transcription luciferase reporter constructs were transiently transfected into the indicated cells, and luciferase activity was analyzed after 48 hours. **F.** The mRNA expression of STAT3 downstream genes cyclin D1, survivin and VEGF was detected in indicated transfected OS cells by qRT-PCR. Data represent the mean ± SD of 3 separate determinations. *p < 0.05, **p < 0.01, ***p < 0.001 by Student's t test.

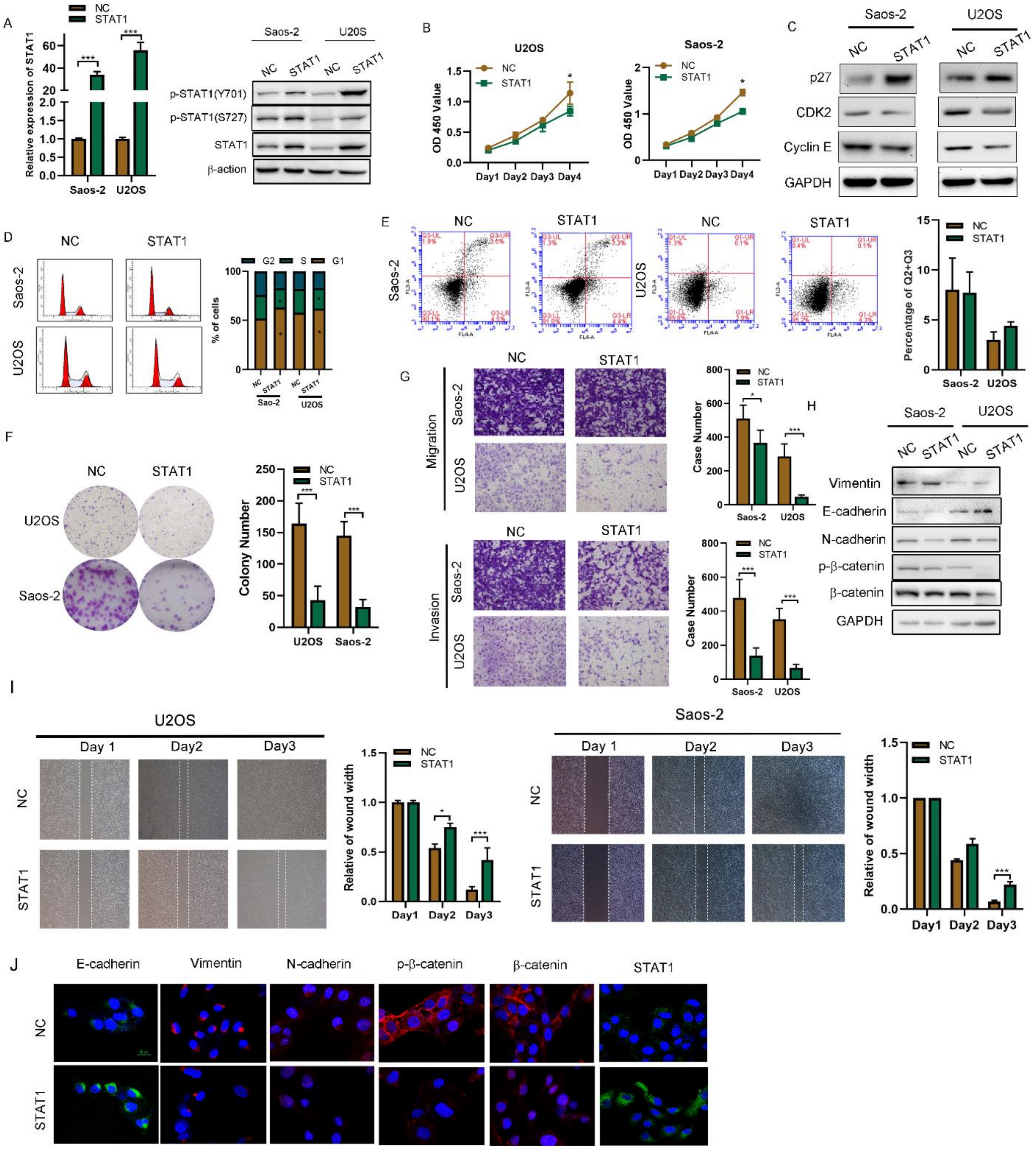

Figure S5. The function of STAT1 in OS cells. **A.** The effect of STAT1 overexpression in U2OS and Saos-2 cells was determined by qRT-PCR and western blot analysis. **B**. A Cell Counting Kit-8 assay was performed on control and STAT1 overexpression U2OS and Saos-2 cells. **C**. The expression of cell cycle biomarkers was detected in control and STAT1-overexpressing OS cells by westernblot. **D**. Cell cycle was detected on control and STAT1-overexpressing OS cells using flow cytometry. **E**. Cell apoptosis was detected by Annexin V-PI stain assay. **F.** Colony formation assays were performed on control and STAT1-overexpressing OS cells. **G**. Transwell assay was performed on control and

STAT1-overexpressing cells to detect the invasion and migration ability. **H.** The expression of EMT biomarkers was detected in STAT1 overexpression OS cells by westernblot. **I.** Wound healing assay was performed in STAT1 overexpression or control OS cell. **J.** The expression of EMT biomarkers was detected by immunofluorescence. Data represent the mean ± SD of 3 separate determinations (Scale bars: 50 μm). *$p < 0.05$, **$p < 0.01$, ***$p < 0.001$ by Student's t test.

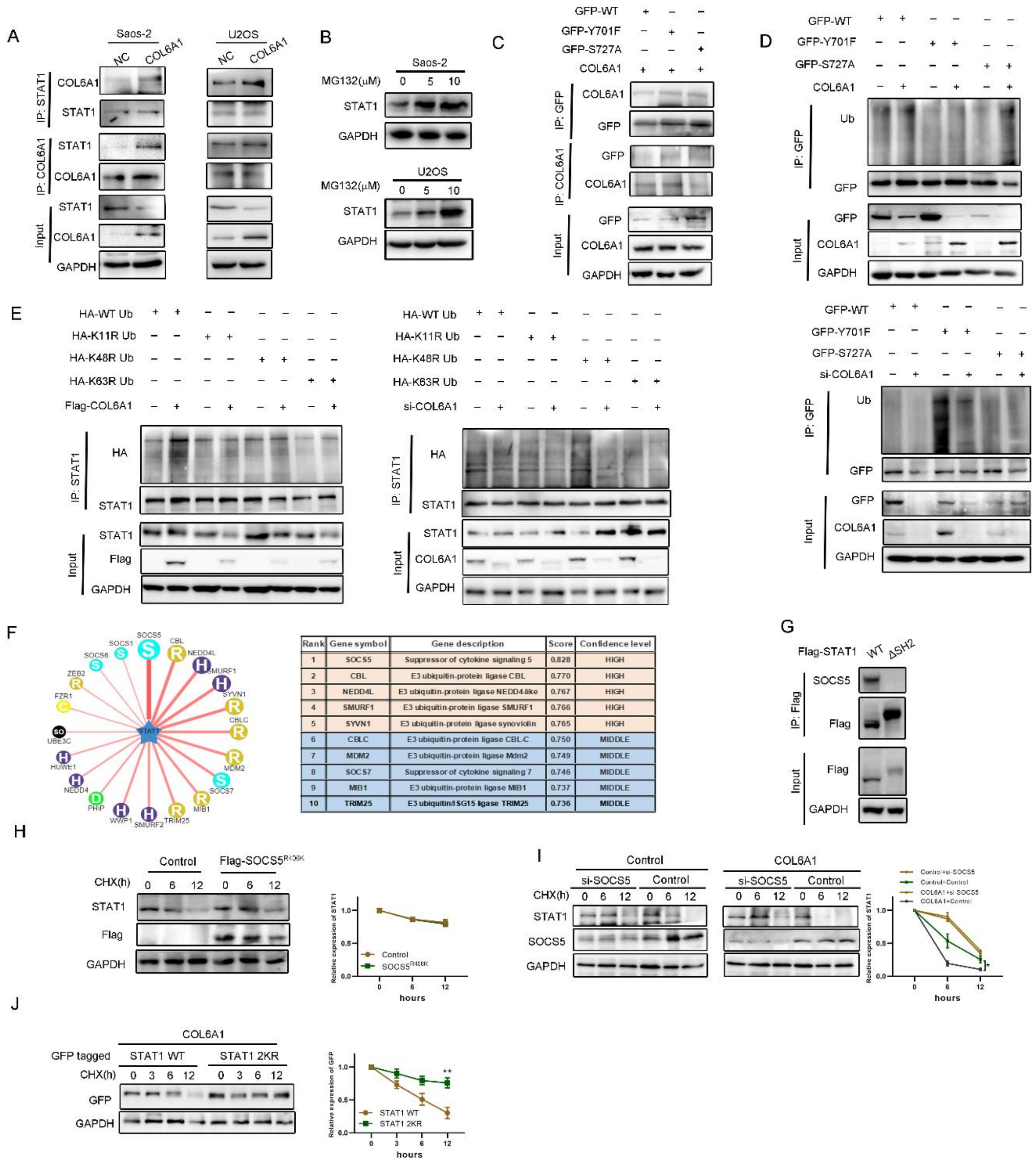

Rank	Gene symbol	Gene description	Score	Confidence level
1	SOCS5	Suppressor of cytokine signaling 5	0.828	HIGH
2	CBL	E3 ubiquitin-protein ligase CBL	0.770	HIGH
3	NEDD4L	E3 ubiquitin-protein ligase NEDD4-like	0.767	HIGH
4	SMURF1	E3 ubiquitin-protein ligase SMURF1	0.766	HIGH
5	SYVN1	E3 ubiquitin-protein ligase synoviolin	0.765	HIGH
6	CBLC	E3 ubiquitin-protein ligase CBL-C	0.750	MIDDLE
7	MDM2	E3 ubiquitin-protein ligase Mdm2	0.749	MIDDLE
8	SOCS7	Suppressor of cytokine signaling 7	0.746	MIDDLE
9	MIB1	E3 ubiquitin-protein ligase MIB1	0.737	MIDDLE
10	TRIM25	E3 ubiquitin/ISG15 ligase TRIM25	0.736	MIDDLE

Figure S6. The ubiquitination of STAT1 mediated with COL6A1. A. The interaction of endogenous COL6A1 and STAT1 was investigated by using co-immunoprecipitation experiments in Saos-2 and

U2OS cells treated with or without COL6A transfection. Co-immunoprecipitation was carried out using anti-STAT1, or anti-COL6A1 antibody. **B**. Saos-2 and U2OS cell lines were treated in the presence of 0-10 μM MG132 for 24 h. Total cell lysates were then prepared for immunoblot detection of STAT1 and β-actin. **C.** GFP-STAT1 (WT), GFP-STAT1 (Y701F), or GFP-STAT1 (S727A) were transfected into Saos-2 cells. Co-immunoprecipitation was performed using GFP antibody and immunoblotting was done using an anti-COL6A1 antibody. **D**. GFP-STAT1 (WT), GFP-STAT1 (Y701F), or GFP-STAT1 (S727A) were transfected into Saos-2 cells together with COL6A1 plasmid or siRNA. STAT1 ubiquitination was detected by immunoprecipitation with anti-GFP antibody and immunoblotting with anti-Ub antibody. **E**. HA-WT, HA-K11R, HA-K48R and HA-K63R were transfected into Saos-2 with COL6A1 or si-COL6A1 transfection. Similar results were observed in three independent experiments. **F**. SOCS5 is predicted as the specific E3 ligase of STAT1 by UbiBrowser database. **G**. Flag-wide type and SH2 domain deletion plasmids were transfected into U2OS. STAT1Co-immunoprecipitation was performed using Flag antibody and immunoblotting was done using an anti-SOCS5 antibody. **H**. The cells expressing mutant SOCS5 were treated with cycloheximide (CHX, 200 μg/mL). The protein levels of STAT1 and Flag were analyzed by western blot. **I**. The cells with indicated treatment were treated with CHX (200 μg/mL). The protein levels of STAT1 and SOCS5 were analyzed by western blot. **J**. The cells with indicated transfection were treated with CHX (200 μg/mL). The protein levels of GFP were analyzed by western blot.

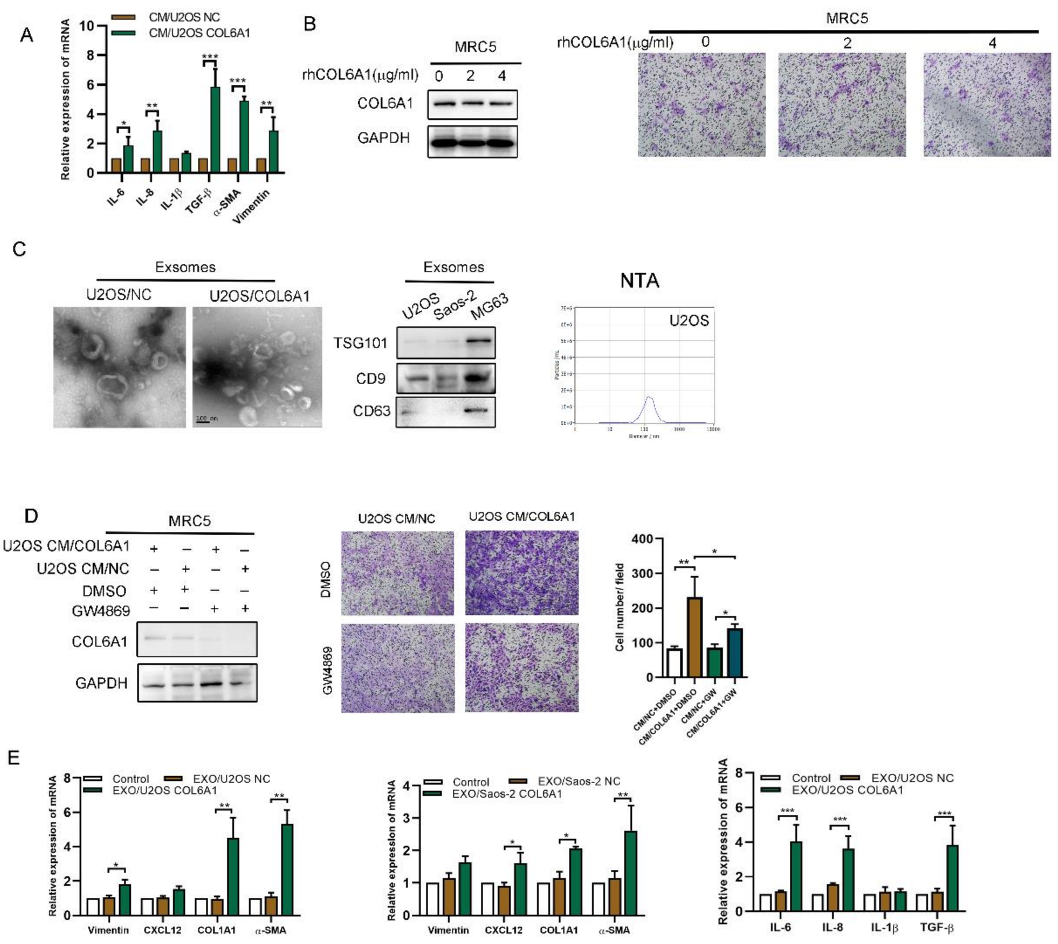

Figure S7. The exosomal COL6A1 derived from OS cells. A. Indicated gene expression of MRC5 treated with CM by COL6A1-transfection OS cells or blank control were detected by qRT-PCR analysis. **B.** The expression of COL6A1 in MRC5 cells detected by westernblot after treated with different concentration of rhCOL6A1 (left panel). The migration ability of MRC5 cells was detected by transwell assay after treated with different concentration of rhCOL6A1(right panel). **C.** Exosomes released by U2OS/NC and U2OS/COL6A1 cells were detected by electron microscopy, Immunoblotting assay and Nanoparticle Tracking Analysis (NTA) (Scale bar:100 nm). **D.** COL6A1 expression was detected in parental OS cells treated with exosomes derived from CM/COL6A1 and CM/NC incubated with DMSO or GW4869 (left panel). Cell migration potential was determined in MRC5 cells incubated with DMSO or GW4869, upon treatment with CM/COL6A1 and CM/NC (right panel). **E.** The mRNA expression of indicated gene was detected in MRC5 treated with the exosomes derived from OS cells. Data represent the mean ± SD of 3 separate determinations. $*p < 0.05$, $**p < 0.01$, $***p < 0.001$ by Student's t test.

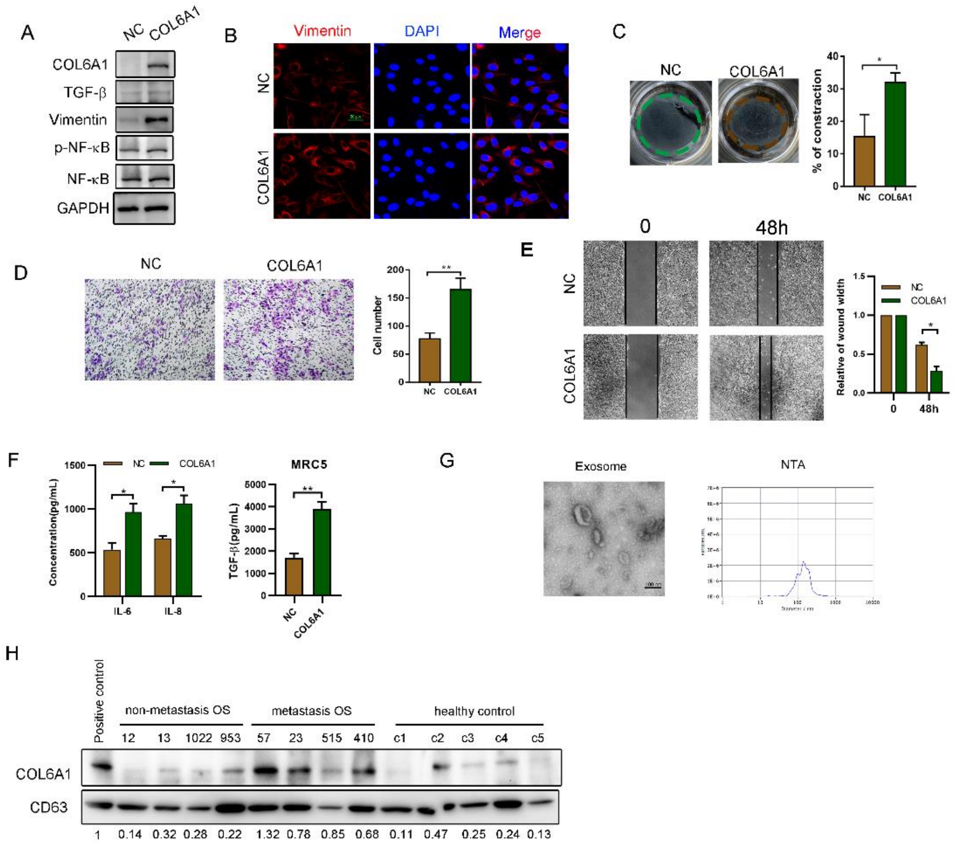

Figure S8. Function of COL6A1 in MRC5 cells. A. The expression of indicated protein was detected by westernblot after COL6A1 transfection in MRC5 cells. **B.** The expression of Vimentin was detected by immunofluorescence in control and COL6A1 transfection MRC5 cells (Scale bars: 50 μm). **C.** MRC5 cells transfected with COL6A1 or control were assessed for their ability to contract collagen. Collagen contraction was quantified by the ImageJ software. **D**. The migration ability of control and COL6A1 transfection MRC5 cells was detected by transwell assay and wound healing assay. **E.** The secretory protein level of IL-6, IL-8 and TGF-β was detected by ELISA. **F.** The rate of lung metastasis after tail-vein injection indicated treatment cells. **G.** Exosomes released by different cancer cells were detected by electron microscopy and Nanosight particle tracking analysis (Scale bar: 200 nm). **H.** The expression of COL6A1 was detected in exosomes derived from healthy people and OS patient's serum. COL6A1 transfected cell protein was used as positive control. Data represent the mean ± SD of 3 separate determinations. Scale bars: 100 μm. *$p < 0.05$, **$p < 0.01$, ***$p < 0.001$ by Student's t test.

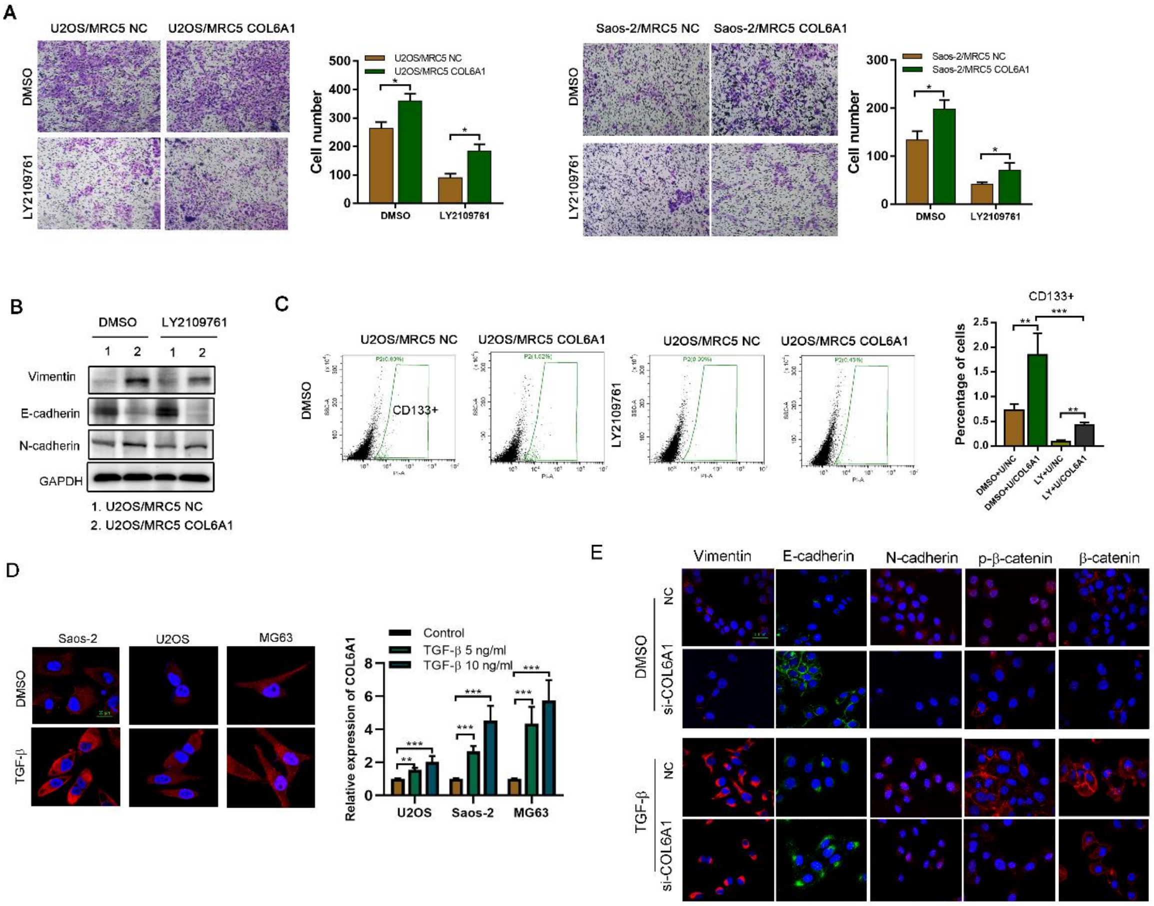

Figure S9. The activated CAF secreted TGF-β. A. The migration of OS cells with indicated treatment were detected by the transwell assay. **B.** The expression of EMT biomarkers was detected by westernblot in the indicated cell treated with TGF-β inhibitor LY2109761 (50 nM). **C.** The percentage of CD133 positive cells was determined by fluorescence-activated cell sorting analysis in indicated cells treated with TGF-β inhibitor LY2109761 (50 nM). **D.** The expression of COL6A1 in OS cells treated with TGF-β was detected by immunofluorescence and qRT-PCR (Scale bars: 20 μm). **E.** Confocal microscope assay was performed to detect the expression of indicated protein expression in siCOL6A1or control U2OS cells treated with TGF-β (Scale bars: 50 μm). Data represent the mean ± SD of 3 separate determinations. *$p < 0.05$, **$p < 0.01$, ***$p < 0.001$ by Student's t test.

Table S1. Clinical parameters of 181 OS patients

NO.	Gender	Age	Group	Size	Group	Location	Stage	Meta	Follow	Status	IHC Score	Group
1	F	9	1	0.3	1	scapula	3	1	11	1	12	1
2	M	16	1	1	1	scapula	1	1	28	1	9	1
3	M	13	1	1	1	scapula	3	1	7	1	8	1
4	M	10	1	1	1	scapula	2	1	3	1	6	2
5	F	18	1	1.5	1	scapula	2	0	16	0	4	2
6	M	11	1	1.5	1	scapula	1	0	42	0	12	1
7	M	10	1	1.7	1	scapula	3	0	19	0	12	1
8	M	20	1	2	1	scapula	3	0	24	0	6	2
9	F	16	1	2	1	humerus	2	0	0	0	6	2
10	F	16	1	2	1	humerus	3	0	1	0	4	2
11	M	10	1	2	1	calcaneus	3	1	5	1	12	1
12	F	6	1	2	1	ilium	2	1	7	1	6	2
13	F	15	1	2.5	1	ilium	2	1	19	1	12	1
14	F	12	1	1.5	1	ilium	1	1	6	1	6	2
15	M	9	1	1	1	rib	2	0	35	0	6	2
16	M	9	1	2.3	1	rib	3	0	12	0	6	2
17	F	8	1	2.6	1	ulna	1	0	16	0	8	1
18	M	8	1	2.5	1	pubis	2	0	34	0	4	2
19	M	18	1	2.6	1	pubis	1	0	4	0	3	2
20	M	14	1	2.1	1	clavicle	3	0	17	0	12	1
21	M	13	1	2.0	1	clavicle	3	0	43	0	6	2
22	F	10	1	1.5	1	NA	3	0	21	0	8	1
23	F	31	2	1.5	1	NA	3	0	4	0	12	1
24	M	50	2	9.6	1	Femur	2	1	3	1	12	1
25	F	57	2	1.6	1	Femur	3	0	19	0	12	1
26	F	46	2	1.4	1	Femur	3	0	11	0	12	1
27	F	55	2	0.9	1	Femur	1	0	1	0	0	2
28	M	81	2	1.3	1	Femur	1	0	6	0	8	1
29	M	26	2	0.8	1	Femur	1	0	17	0	8	1
30	F	35	2	0.5	1	Femur	1	0	6	0	4	2
31	M	50	2	0.6	1	Femur	1	0	3	1	12	1
32	F	46	2	2.5	1	Femur	3	0	3	1	12	1
33	F	54	2	2.5	1	Femur	3	0	9	1	8	1
34	F	52	2	1	1	Femur	2	0	35	0	4	2
35	M	45	2	1	1	Femur	1	0	1	0	8	1
36	M	42	2	1.5	1	Femur	2	0	22	0	12	1
37	F	47	2	2.2	1	Femur	3	0	40	1	12	1
38	F	36	2	2.4	1	Femur	2	0	22	1	9	1
39	F	65	2	1	1	Femur	3	0	4	0	12	1
40	M	59	2	2.3	1	Jawbone	3	0	16	0	8	1
41	M	36	2	1	1	Jawbone	3	1	7	1	12	1
42	M	44	2	1	1	Jawbone	3	1	3	1	8	1
43	F	47	2	2.4	1	Jawbone	1	0	2	0	3	2
44	M	46	2	1	1	Jawbone	3	0	36	0	6	2
45	F	43	2	2.5	1	Jawbone	1	0	50	0	8	1
46	M	28	2	1	1	Jawbone	3	0	38	0	12	1
47	M	27	2	1	1	Jawbone	1	0	21	0	12	1
48	M	25	2	1.5	1	Tibia	3	0	12	0	12	1
49	M	53	2	2.8	1	Tibia	3	0	6	0	3	2
50	M	26	2	0.5	1	Tibia	3	0	24	0	12	1
51	F	34	2	2.8	1	Tibia	2	0	2	0	8	1
52	F	58	2	1.6	1	Tibia	3	0	19	0	12	1
53	M	43	2	2.7	1	Tibia	3	0	19	0	4	2
54	F	13	1	2.5	1	Tibia	2	0	4	0	8	1
55	M	15	1	3	1	Tibia	2	0	24	0	6	2
56	M	22	1	1.4	1	Tibia	2	0	12	0	12	1
57	F	13	1	2.7	1	Tibia	3	0	3	0	8	1
58	M	13	1	1.9	1	Tibia	1	0	43	0	8	1
59	M	20	1	2.5	1	Tibia	2	0	58	0	12	1
60	F	9	1	2.5	1	Tibia	2	0	5	0	6	2

61	F	20	1	2.0	1	Tibia	2	1	2	1	9	1
62	M	13	1	3.5	2	scapula	1	1	8	1	12	1
63	F	14	1	4	2	scapula	3	0	5	0	6	2
64	M	21	1	4	2	scapula	3	0	9	0	6	2
65	M	15	1	4.5	2	scapula	2	0	23	0	4	2
66	F	18	1	4.5	2	scapula	2	0	2	0	9	1
67	M	7	1	5	2	humerus	1	0	9	0	4	2
68	F	12	1	5.5	2	humerus	3	0	8	0	12	1
69	F	16	1	6	2	humerus	1	0	2	0	6	2
70	F	8	1	6	2	humerus	3	0	19	0	8	1
71	M	11	1	6	2	humerus	3	0	15	0	8	1
72	M	8	1	6	2	humerus	1	0	9	0	2	2
73	M	9	1	6	2	calcaneus	1	0	1	0	3	2
74	F	8	1	7	2	calcaneus	1	1	2	1	8	1
75	F	12	1	7	2	calcaneus	3	1	6	1	9	1
76	M	21	1	10	2	calcaneus	2	1	21	1	8	1
77	M	13	1	10	2	ilium	3	1	6	1	0	2
78	M	11	1	10.6	2	ilium	3	1	19	1	12	1
79	F	17	1	7	2	rib	2	1	10	1	12	1
80	M	14	1	8	2	rib	2	1	14	1	6	2
81	M	14	1	8	2	rib	3	0	16	1	12	1
82	M	14	1	8	2	rib	3	0	10	0	4	2
83	M	9	1	5	2	ulna	3	0	49	0	8	1
84	F	20	1	4	2	ulna	1	0	15	0	2	2
85	F	17	1	13	2	ulna	3	0	13	0	12	1
86	F	17	1	6	2	pubis	3	0	9	0	0	2
87	F	12	1	7	2	pubis	2	0	1	0	8	1
88	M	13	1	7	2	clavicle	3	0	7	0	8	1
89	F	20	1	8	2	NA	3	0	44	0	12	1
90	F	14	1	5	2	NA	3	0	4	0	12	1
91	M	13	1	6	2	NA	3	0	8	0	2	2
92	M	13	1	8.5	2	Femur	3	1	6	1	4	2
93	F	21	1	6	2	Femur	3	1	16	1	12	1
94	F	15	1	12	2	Femur	3	0	18	0	6	2
95	M	15	1	6	2	Femur	2	0	8	0	2	2
96	F	31	2	6	2	Femur	3	0	2	0	3	2
97	F	34	2	10	2	Femur	3	0	4	0	8	1
98	M	47	2	4.5	2	Femur	2	0	14	0	6	2
99	F	30	2	7.5	2	Femur	2	0	22	0	12	1
100	M	46	2	4	2	Femur	1	0	2	0	4	2
101	M	40	2	7	2	Femur	2	0	16	0	4	2
102	M	62	2	5	2	Femur	3	0	5	0	8	1
103	M	52	2	7	2	Femur	2	0	13	0	1	2
104	M	53	2	4	2	Femur	2	0	3	0	2	2
105	M	32	2	4.2	2	Femur	3	0	15	0	6	2
106	M	40	2	3	2	Femur	2	0	4	0	1	2
107	M	33	2	4	2	Femur	3	0	50	0	6	2
108	M	40	2	4.5	2	Femur	2	0	3	0	4	2
109	F	33	2	3	2	Femur	3	0	22	1	8	1
110	M	28	2	3	2	Femur	2	0	12	1	4	2
111	M	31	2	6	2	Femur	1	0	18	1	6	2
112	M	49	2	4.8	2	Femur	3	0	5	1	8	1
113	F	7	1	3.5	2	Femur	3	0	12	1	8	1
114	M	16	1	5	2	Femur	3	0	5	1	12	1
115	F	66	2	3.5	2	Femur	3	0	20	1	8	1
116	M	27	2	6	2	Femur	2	0	10	1	0	2
117	F	66	2	4	2	Femur	1	0	10	0	8	1
118	F	10	2	2	2	Femur	3	0	23	0	6	2
119	M	44	2	4	2	Femur	3	0	7	0	2	2
120	M	45	2	7	2	Femur	3	0	10	0	3	2

121	F	34	2	4	2	Femur	2	0	3	0	8	1
122	M	34	2	4.8	2	Femur	3	0	2	0	12	1
123	M	37	2	11	2	Femur	3	0	15	1	8	1
124	F	34	2	8	2	Femur	1	0	20	1	8	1
125	M	33	2	4.5	2	Femur	3	0	67	1	8	1
126	F	32	2	5	2	Femur	3	0	23	1	6	2
127	M	31	2	8	2	Femur	3	0	6	1	12	1
128	F	31	2	7.4	2	Femur	1	0	13	1	12	1
129	M	30	2	6	2	Femur	3	0	22	1	8	1
130	M	43	2	3.5	2	Femur	2	0	3	1	12	1
131	M	38	2	5	2	Femur	3	0	7	0	2	2
132	M	30	2	4.8	2	Femur	1	0	8	0	8	1
133	M	28	2	5	2	Femur	3	0	13	0	4	2
134	F	26	2	4.8	2	Femur	1	0	0	0	8	1
135	F	45	2	5.8	2	Jawbone	3	0	3	0	8	1
136	F	54	2	4.6	2	Jawbone	3	1	11	1	4	2
137	M	58	2	6	2	Jawbone	1	1	2	0	12	1
138	M	79	2	8	2	Jawbone	2	0	1	0	0	2
139	F	12	1	8.5	2	Jawbone	3	0	18	0	4	2
140	M	16	1	8	2	Jawbone	3	0	3	1	4	2
141	F	14	1	5	2	Jawbone	3	0	3	0	4	2
142	M	16	1	9	2	Jawbone	3	0	4	0	4	2
143	M	12	1	5	2	Jawbone	1	0	17	0	9	1
144	M	13	1	7	2	Jawbone	3	0	4	0	2	2
145	M	18	1	5	2	Jawbone	3	0	9	0	4	2
146	M	20	1	5	2	Jawbone	3	0	10	0	12	1
147	F	9	1	12	2	Jawbone	1	0	15	0	2	2
148	F	18	1	3	2	Jawbone	3	0	4	0	8	1
149	F	17	1	14	2	Jawbone	3	0	4	0	12	1
150	F	16	1	11	2	Jawbone	2	0	5	0	4	2
151	M	16	1	6	2	Jawbone	2	0	4	0	8	1
152	M	16	1	6	2	Jawbone	3	0	21	0	2	2
153	M	16	1	8	2	Tibia	1	1	2	1	12	1
154	F	15	1	6	2	Tibia	3	1	10	1	4	2
155	M	14	1	8	2	Tibia	3	1	2	1	8	1
156	M	13	1	8	2	Tibia	2	0	17	0	4	2
157	F	13	1	7	2	Tibia	3	0	1	0	4	2
158	M	12	1	7	2	Tibia	3	0	4	0	4	2
159	F	12	1	9	2	Tibia	2	0	9	0	8	1
160	F	12	1	4.8	2	Tibia	1	0	3	0	6	2
161	M	11	1	5	2	Tibia	2	0	4	0	4	2
162	F	10	1	6	2	Tibia	2	0	10	1	12	1
163	M	9	1	4.9	2	Tibia	1	0	24	0	9	1
164	M	8	1	4.8	2	Tibia	3	0	4	0	6	2
165	F	8	1	8.5	2	Tibia	2	0	22	0	2	2
166	F	14	1	6.5	2	Tibia	2	0	7	0	1	2
167	M	13	1	5.4	2	Tibia	3	0	3	0	6	2
168	F	22	1	4.7	2	Tibia	1	0	22	0	8	1
169	F	19	1	4.5	2	Tibia	3	0	3	0	1	2
170	F	19	1	4	2	Tibia	3	0	12	0	8	1
171	F	19	1	6	2	Tibia	3	0	10	0	12	1
172	F	18	1	12	2	Tibia	3	0	4	0	2	2
173	F	14	1	9.4	2	Tibia	1	0	2	0	12	1
174	M	60	2	5	2	Tibia	1	0	4	0	8	1
175	F	43	2	8.5	2	Tibia	3	1	2	1	12	1
176	F	51	2	7	2	Tibia	2	1	18	0	4	2
177	M	33	2	5	2	Tibia	1	0	26	0	4	2
178	F	53	2	6.8	2	Tibia	3	0	1	0	4	2
179	F	29	2	7.5	2	Tibia	3	0	11	0	2	2
180	F	39	2	7	2	Tibia	3	0	4	0	4	2
181	M	26	2	7.5	2	Tibia	3	0	21	0	6	2

119

Table S2. Sequence of primers

COL6A1	F: 5’-TCAAGAGCCTGCAGTGGATG-3’; R: 5’-TGGACACTTCTTGTCTATGCAG-3’
GAPDH	F:5’-AGAAGGCTGGGCTCATTTG-3’; R: 5’-AGGGGCCATCCACAGTCTTC-3’
IL-1β	F: 5'-ATGATGGCTTATTACAGTGGCAA-3'; R: 5'-GTCGGAGATTCGTAGCTGGA-3'
IL-6	F: 5'-ACTCACCTCTTCAGAACGAATTG-3'; R: 5'-CCATCTTTGGAAGGTTCAGGTTG-3'
IL-8	F: 5'-TTTTGCCAAGGAGTGCTAAAGA-3'; R: 5'-AACCCTCTGCACCCAGTTTTC-3'
CD133	F: 5'-GCCACCGCTCTAGATACTGC-3’; R: 5'-TGTTGTGATGGGCTTGTCAT-3'
E-cadherin	F: 5'-TGCCCAGAAAATGAAAAAGG-3'; R: 5'-GTGTATGTGGCAATGCGTTC-3'
Vimentin	F: 5'-GAGAACTTTGCCGTTGAAGC-3’; R: 5'-GCTTCCTGTAGGTGGCAATC-3'
N-cadherin	F: 5'- ACCAGCCTCCAACTGGTAT-3'; R: 5'-TACGACGTTAGCCTCGTTC-3'
STAT1	F: 5'-CAGCTTGACTCAAAATTCCTGGA-3'; R: 5'-TGAAGATTACGCTTGCTTTTCCT-3'
STAT3	F: 5'-CAGCAGCTTGACACACGGTA-3’; R: 5'-AAACACCAAAGTGGCATGTGA-3'
CXCL12	F: 5'-ATTCTCAACACTCCAAACTGTGC; R: 5'-ACTTTAGCTTCGGGTCAATGC-3'
COL1A1	F: 5'-GAGGGCCAAGACGAAGACATC-3'; R: 5'-CAGATCACGTCATCGCACAAC-3'
COL3A1	F:5'-GGAGCTGGCTACTTCTCGC-3'; R: 5'-GGGAACATCCTCCTTCAAACAG-3'
FOSB	F: 5'-GCTGCAAGATCCCCTACGAAG-3’; R: 5'-ACGAAGAAGTGTACGAAGGGTT-3'
EPHB1	F: 5'-GGCTGCGATGGAAGAAACG-3’; R: 5'-CTGGTTGGGCTCGAAGACATT-3'
BEST1	F: 5'-CTGGGCTTCTACGTGACGC-3’; R: 5'-TTGCTCGTCCTTGCCTTCG-3'
HSPA1A	F: 5'-AGCTGGAGCAGGTGTGTAAC-3'; R: 5'-CAGCAATCTTGGAAAGGCCC-3'
P300	F: 5'GACCCTCAGCTTTTAGGAATCC-3'; R: 5'-TGCCGTAGCAACACAGTGTCT-3'
TGF-β	F: 5'-GGGCATGTGGCTTCTATGGT-3'; R: 5'-CCCCAAGCGCATCTCGTAG-3'
COL6A1 promoter1	F: 5'-GACACGCTGGTTTTCAGACG-3'; R: 5'-GCAGTTCAGTTCCCGTGTCA-3'
COL6A1 promoter2	F: 5'-AACAATTCAGCTCACCGGCGA-3'; R: 5'-ACCCAGGGAGAGTTCCTTGA-3'
Cyclin D1	F:5'-GGATGCTGGAGGTCTGCGA -3’; R: 5'-TAGAGGCCACGAACATGCAAG-3'
Survivin	F:5'-CAAGGAGCTGGAAGGCTGG-3’; R:5'-GTTCTTGGCTCTTTCTCTGTCC-3'
VEGF	F:5’-AAAGGAGCCTACAAGA-3’; R:5'-TTCACAAGCAGCCAAT-3'
